# Effectiveness of Virtual Reality–Based Early Rehabilitation Strategies on Pain, Sleep, Anxiety, Balance, Cognition, and Limb Motor Function in Adult Intensive Care Unit Patients: Systematic Review and Meta-Analysis of Randomized Controlled Trials

**DOI:** 10.2196/81865

**Published:** 2026-03-06

**Authors:** Fei Wu, Yunting Wu, Yana Xing, Weixin Cai, Ran Zhang

**Affiliations:** 1 Nursing Department, Beijing Tiantan Hospital, Capital Medical University, No. 119, South 4th Ring West Road, Fengtai District, Beijing, China, Beijing, Beijing, China, 86 59978336; 2 School of Nursing, Capital Medical University, Beijing, Beijing, China; 3 Department of Critical Care Medicine, Beijing Tiantan Hospital, Capital Medical University, Beijing, Beijing, China

**Keywords:** virtual reality, intensive care unit, early rehabilitation, randomized controlled trial, systematic review, meta-analysis

## Abstract

**Background:**

Early rehabilitation is vital for functional recovery in critically ill patients. Virtual reality–based early rehabilitation intervention (VR-ERI) is an emerging strategy, but evidence on its feasibility, safety, and efficacy remains inconsistent and unsynthesized.

**Objective:**

We synthesized evidence from randomized controlled trials (RCTs) on the feasibility and safety of VR-ERI in adult critically ill patients and evaluated its effects on functional outcomes during intensive care unit (ICU) stay and at short-term follow-up (≤3 months post ICU).

**Methods:**

Following PRISMA (Preferred Reporting Items for Systematic Reviews and Meta-Analyses) 2020 guidelines, we searched 10 databases (eg, PubMed, Web of Science, Cochrane Library, and Embase) from inception to October 5, 2025, for Chinese and English publications. We included RCTs comparing VR-ERI with control measures initiated early (within 72 h of ICU admission) in adults. FW and YW independently screened studies and extracted data; YW and YX independently conducted the revised Cochrane Risk of Bias tool assessment. Data were synthesized narratively or via meta-analysis in R Studio using a random-effects model (Hartung-Knapp-Sidik-Jonkman adjustment). Effects were expressed as standardized mean differences (SMD; with Hedges g correction) with 95% CIs and 95% prediction intervals (95% PIs). Subgroup and sensitivity analyses were performed to explore heterogeneity and assess robustness. The Grading of Recommendations Assessment, Development, and Evaluation framework was used to assess the quality of evidence.

**Results:**

Sixteen RCTs (published 2020‐2025) involving 1356 patients were included. Bias assessment found 1 study at low risk, 5 with some concerns, and 10 at high risk. Meta-analysis suggested potential trends for VR-ERI in improving ICU anxiety (SMD −0.86, 95% CI −1.85 to 0.13, 95% PI −3.75 to 2.03; very low certainty) and subjective sleep quality (SMD 3.36, 95% CI 0.77-5.94; very low certainty), with a more pronounced effect in the Richards-Campbell Sleep Questionnaire–assessed subgroup (SMD 5.12, 95% CI 0.54-9.71). At follow-up, VR-ERI showed trends toward improved balance (Berg Balance Scale: SMD 0.97, 95% CI 0.74-1.20, 95% PI 0.37-1.58; moderate certainty), limb motor function (Fugl-Meyer: SMD 1.40, 95% CI −0.23 to 3.02; low certainty), and cognitive function (SMD 0.78, 95% CI 0.16-1.39; low certainty). No significant differences were found for objective sleep measures or ICU pain (low to very low certainty). No serious adverse events were reported; only a few studies mentioned mild reactions, such as dizziness, nausea, and fatigue.

**Conclusion:**

This review indicates VR-ERI’s potential to improve anxiety, subjective sleep, balance, cognition, and motor function in early ICU rehabilitation, while its effects on pain and objective sleep remain unclear and safety protocols need refinement. Given the high risk of bias, substantial heterogeneity, and imprecision, the overall certainty of evidence is low. Thus, VR-ERI may serve as a nonpharmacological adjunct, but its clinical translation requires consideration of cost and patient suitability, and more rigorous research is needed.

## Introduction

### Background

Patients in the intensive care unit (ICU) often experience a series of physical, cognitive, and psychological impairments after discharge due to factors such as physiological dysfunction, surgical trauma, pain, and reduced social interaction [1]. In 2010, the Society of Critical Care Medicine first introduced the term “post-intensive care syndrome (PICS)” to define this phenomenon [2,3]. The manifestations commonly include weakness, pain, anxiety, sleep disturbances, and cognitive dysfunction, which can persist for months or even years. Studies indicate that 47.9% of patients still exhibit cognitive impairment 3‐6 months after transfer from the ICU [4]; 68% (355/523) report persistent pain after discharge, with pain intensity remaining at a moderate level even 1 year later [5]. Additionally, 55% of patients experience sleep disturbances within the first month after discharge [6]. Furthermore, 12.3% (98,530/799,645) of critically ill patients are diagnosed with depression or anxiety after discharge, and these patients demonstrate higher long-term mortality rates [7]. PICS severely compromises patients’ quality of life, reduces functional independence, and imposes a significant care burden on families and society [8].

To reduce long-term impairments after critical illness, the Society of Critical Care Medicine recommends that health care professionals assess patients and coordinate care strategies for injury management beginning at ICU admission [9]. As clinical treatment advances, early functional recovery has become a key focus for preventing PICS [10]. Early rehabilitation is generally initiated within 72 hours of ICU admission, following achievement of hemodynamic and respiratory stability (typically within 24‐48 h) [11,12]. It commonly includes early physical activity [13,14], neurocognitive stimulation [15,16], psychological intervention [17], and multimodal programs [18]. Existing meta-analyses confirm that routine early rehabilitation started within 72 hours may improve physical and cognitive function, help prevent PICS without increasing adverse events [19], and reduce the incidence of ICU-acquired weakness [20]. However, no differences have been found in cognitive-related delirium-free days or Hospital Anxiety and Depression Scale scores. Another meta-analysis on nurse-led early rehabilitation showed it significantly shortens ICU length of stay, although effects on functional outcomes, such as mobility, muscle strength, and mortality, were not significant [21]. These inconsistencies may relate to factors including the time-intensive nature of current measures, insufficient standardization in implementation, and low patient participation and adherence.

Virtual reality (VR), a term first introduced by computer scientist Jaron Lanier in 1989, refers to a simulation technology providing users with an immersive environmental experience [22]. VR technology integrates computer, electronic information, and simulation technologies to enable users to interact with the VR system and experience an immersive, tactile, visual, and multisensory environment via VR helmets, all-in-one devices, and other gadgets [23]. The system is known for its novelty, interactivity, and immersive qualities [24]. VR technology–based rehabilitation techniques have been widely adopted in clinical rehabilitation. There is evidence suggesting that these techniques can effectively improve cognitive function in patients with stroke [25], reduce pain levels in patients with chronic low back pain and neck pain [26,27], and alleviate anxiety and depression [28,29].

Virtual reality–based early rehabilitation intervention (VR-ERI) is an emerging ICU recovery strategy that is gaining attention and has shown preliminary application results. Using VR technology, it provides ICU patients with specific virtual environments, such as natural landscapes [30], meditation music [31], or virtual tasks (such as memory games [32]), and cognitive exercises like “catching coconuts” and “avoiding obstacles” [33]. This aims to enhance rehabilitation motivation, participation, and physiological and psychological function. Its interactive, immersive, and feedback-driven design makes VR-ERI a promising tool for personalized early rehabilitation in critically ill patients. However, current research findings are inconsistent. Some studies report that VR-ERI effectively reduces anxiety [32], while others show no significant difference [34,35]. Such discrepancies may affect the certainty and consistency of clinical decision-making, potentially hindering the technology’s wider adoption in ICU early rehabilitation.

To our knowledge, researchers [36] have explored the interventional effects of VR therapy on patients’ PICS through a meta-analysis, but its included population did not specifically focus on early rehabilitation strategies during the ICU period. This analysis pooled data from patients receiving VR therapy both within the ICU and during follow-up after discharge. Due to differences between these 2 populations in aspects such as intervention timing and environment, combining them in the analysis may introduce clinical heterogeneity, thereby weakening the credibility of the results. Another meta-analysis indicated that compared with traditional rehabilitation, VR-based early motor rehabilitation can improve balance, functional walking, and upper and lower limb motor function in critically ill patients [37]. However, this review only assessed motor function and did not comprehensively examine the effects on other early rehabilitation outcome indicators.

### Objectives

This systematic review and meta-analysis aim to (1) characterize the types and content of VR-ERI activities and analyze the feasibility and safety of VR technology implementation in critically ill patients; (2) systematically evaluate the effectiveness of VR-ERI on physiological, psychological, and cognitive outcomes during ICU treatment and at short-term follow-up (within 3 months post-ICU discharge); and (3) provide recommendations for future research.

## Methods

### Study Protocol and Registration

The review protocol was registered in PROSPERO. This systematic review was conducted in accordance with the PRISMA (Preferred Reporting Items for Systematic Reviews and Meta-Analyses) 2020 statement [38] to ensure transparency (Checklists 1-3).

### Deviations From the PROSPERO Protocol

During implementation, the search language was limited to Chinese and English to ensure assessment accuracy, and the search cutoff date was extended to October 5, 2025, to incorporate the latest evidence. Regarding statistical analysis, due to heterogeneity among the included studies, all analyses adopted the more conservative random-effects model. To enhance statistical robustness, the analysis was switched to the R language (version 4.4.1; R Core Team), and the Hartung-Knapp-Sidik-Jonkman method was applied to correct random-effects outcomes. This method provides more conservative CIs when the number of studies is small, reducing the risk of false-positive results in the presence of high heterogeneity. Apart from the above adjustments, all other procedures remained consistent with the original protocol.

### Data Sources and Search Strategy

Two independent researchers (FW and YW) conducted literature searches across 6 English-language and 4 Chinese-language databases. MEDLINE and Embase were searched simultaneously via the Ovid platform. The other English databases were searched on their respective platforms—PubMed (via National Center for Biotechnology Information), Web of Science (via Clarivate Analytics), the Cochrane Library, and PsycINFO (via ProQuest). The Chinese databases searched were China National Knowledge Infrastructure (CNKI), WanFang Data, VIP Database (VIP), and the SinoMed Service System (SinoMed). The search strategy combined Medical Subject Headings with free-text terms using Boolean operators. The search was performed in 2 stages. An initial search covered records from the inception of each database until May 11, 2025, and to incorporate the most recent evidence, the full search strategy was rerun on October 5, 2025, extending the cutoff date accordingly. All searches were limited to Chinese and English publications to align with the team’s language capabilities and ensure feasibility.

All search strategies were independently designed based on the research question and were peer-reviewed and refined by experts in evidence-based medicine and critical care medicine in accordance with the PRISMA-S (Preferred Reporting Items for Systematic reviews and Meta-Analyses literature search extension) checklist [39]. No published search filters were used, nor were search strategies from other systematic reviews adapted or reused. To cover the literature as comprehensively as possible, we also used the snowballing method to trace references and citations and attempted to obtain full-text articles by contacting librarians or the original authors. Furthermore, as this study focused on published efficacy evidence, we did not specifically search clinical trial registries or gray literature (including conference proceedings). The complete search strategies and procedures are detailed in Multimedia Appendix 1.

### Study Selection

The rationale for formulating inclusion and exclusion criteria was based on the Population, Intervention, Comparison, Outcome, and Study Design (PICOS) framework, (1) population: adults aged ≥18 years receiving treatment during their ICU stay; (2) intervention: VR-ERI; (3) control: no treatment, usual care measures, or non–VR-based interventions; (4) outcomes: studies reporting at least 1 physical, psychological, or cognitive function outcome (such as cognition, pain, delirium, anxiety, depression, and sleep quality) or feasibility and safety outcomes during ICU treatment or after ICU transfer; and (5) study design: randomized controlled trials (RCTs) published in Chinese or English. The exclusion criteria were (1) studies that did not report original outcome measures and for which relevant outcome data could not be obtained after contacting the authors; (2) duplicate publications; and (3) case reports, letters, conference abstracts, and review articles.

### Screening

In total, 2 independent researchers (FW and YW) conducted the literature screening and data extraction, followed by cross-checking. Any uncertainties were resolved by WC. First, duplicate records were automatically identified and removed using the built-in function of the EndNote X9 (Clarivate) reference management software, based on fields such as title, author, publication year, and journal name. Subsequently, the 2 researchers (FW and YW) independently examined the titles, authors, and other key information of the remaining records to further exclude any duplicates not detected by the software. Next, titles and abstracts were reviewed to exclude obviously irrelevant literature. Finally, full texts were retrieved and assessed against the eligibility criteria to determine the final set of included studies.

### Data Extraction

A total of 2 independent researchers (FW and YW) performed data extraction using a uniformly developed standardized form without using automated tools. The data that were systematically extracted were (1) general information, including first author, country, and publication year; (2) participant characteristics, including disease type, sample size, and baseline demographic and clinical characteristics; and (3) intervention characteristics, including specific type of VR, content of the VR-based intervention, content of the control group intervention, intervention duration, intervention frequency, outcome measures, measurement tools, time points of data collection and raw data (such as mean, SD, median, and IQR), for outcomes reported at baseline, post intervention, or follow-up for both groups.

To manage multiple data points objectively and consistently, we predefined selection criteria following the Cochrane Handbook [40]. The core principle was to prioritize data that best aligned with the review’s primary objective, had the highest validity, or greatest clinical representativeness. For multiple measures, we selected the tool that best matched the outcome definition or had the highest validity; for multiple intervention groups, we included the primary intervention group, the most clinically representative, or the largest in sample size; for multiple control groups, we chose the “most representative usual care” group. Where data were missing, unclear, or incomplete, we contacted corresponding authors by email for clarification or missing data. If no response was received after 2 attempts, we used recommended formulas to estimate means and SDs from available data (eg, based on sample size, median, and range) [41].

### Risk-of-Bias Assessment

The second author (YW) and the third author (YX) independently assessed the risk of bias of the included studies using the revised Cochrane Risk of Bias tool (RoB 2) for randomized trials [42]. The RoB 2 tool assesses five domains for randomized trials, which are (1) bias arising from the randomization process, (2) bias due to deviations from the intended interventions, (3) bias due to missing outcome data, (4) bias in the measurement of the outcome, and (5) bias in the selection of the reported result. Each domain is judged against a series of signaling questions. The overall risk of bias for a study is categorized as “low,” “some concerns,” or “high.” A study is judged to have a low overall risk of bias if all domains are rated as “low.” An overall rating of “some concerns” is given if at least 1 domain is rated as having “some concerns” and no domain is rated as “high.” An overall “high” risk of bias is judged if any single domain is rated as “high” or if multiple domains are rated as having “some concerns.”

### Data Synthesis and Analysis

To ensure transparency in the data synthesis process, we followed a systematic decision-making procedure. First, all studies had to meet the predefined PICOS criteria and provide the raw data for the relevant outcome measures. Second, we established a rule that each study would contribute only a single data point for a given outcome. If a study contained multiple measurements or multiple group comparisons, a single dataset was selected for pooling according to the prespecified criteria, aiming to minimize heterogeneity as much as possible.

All analyses were performed using the R language (version 4.4.1) within the R Studio integrated development environment (version 2023.12). The metafor package was used for effect size pooling and forest plot generation [43], providing visual representations of each study’s effect size with its 95% CI, along with the pooled overall effect size. All included outcome measures were continuous variables. We conducted a meta-analysis using change scores (from baseline to postintervention or follow-up) in means and SDs for both groups. For studies reporting only pre- and postintervention means and SDs, the mean and SD of change scores were calculated as Mean_change_=Mean_post_−Mean_pre_ and SD__change_= √ (SD__pre_²+ SD__post_² - 2 × r×SD__pre_×SD__post_), where r represents the correlation coefficient between pre- and postintervention values. When correlations were not reported in the original publications, they were calculated according to the Cochrane Handbook for Systematic Reviews of Interventions. To harmonize different assessment tools and ensure consistent direction of results, we reversed the scoring for subjective sleep quality assessed by the Pittsburgh Sleep Quality Index and cognitive scores assessed by the Perceived Deficits Questionnaire for Depression, following Cochrane Handbook guidelines [40]. Consequently, higher scores in the pooled results indicate better sleep quality and cognitive function in patients. Additionally, we categorized outcome measures by assessment time points, specifically divided into (1) during ICU treatment and (2) short-term follow-up (2 wk to 3 mo after ICU discharge).

Given the variability in assessment tools and the presence of clinical and methodological heterogeneity among the included studies, the standardized mean difference (SMD) was adopted as the effect size measure for meta-analysis outcomes. The adjusted Hedges g was used to estimate the effect sizes [44,45]. Absolute SMD values of 0.2, 0.5, and 0.8 were interpreted as small, medium, and large effect sizes, respectively [46]. A random-effects model was applied to derive more conservative effect estimates. To enhance the robustness of statistical inference, particularly in reducing the risk of false-positive results when the number of studies was limited, the Hartung-Knapp-Sidik-Jonkman adjustment was used during model fitting to estimate the pooled effect size and its 95% CI [47].

To quantify between-study heterogeneity and enhance the generalizability of the results in real-world settings, we used a comprehensive assessment strategy. First, absolute heterogeneity was quantified using *τ*² (between-study variance) and its square root τ (between-study SD) [48]. *τ*² provides an absolute measure of the variation between studies, while τ, being on the same scale as the effect size, facilitates clinical interpretation. Second, the Cochran Q test was used for the hypothesis testing of effect size homogeneity, supplemented by the *I*² statistic to quantify the relative extent of heterogeneity [49], with thresholds of 25%, 50%, and 75% used to define low, moderate, and high levels, respectively [50]. Finally, for outcome measures involving at least 3 studies, 95% prediction intervals (PIs) were calculated [51] to estimate the potential range of the true effect size in future similar interventions, thereby providing more clinically informative guidance for practice.

Subgroup analyses were performed to explore potential sources of heterogeneity. A leave-one-out sensitivity analysis was conducted to assess the robustness of the pooled results. For outcome measures with fewer than 2 included studies, descriptive analyses were performed. Additionally, to assess the potential for small-study effects (such as publication bias), we performed several checks for the anxiety outcome, which had the largest number of included studies (n=8). These included a visual inspection of funnel plot symmetry, the Egger test (regression test), and the Begg test (rank correlation test). Furthermore, the trim-and-fill method was used to estimate the potential number of missing studies and to compute an adjusted effect size, thereby evaluating the potential impact of small-study effects on the pooled result. The threshold for statistical significance was set at *P*≤.05.

### Evidence Quality

To evaluate the reliability of the evidence, we used the Grading of Recommendations Assessment, Development, and Evaluation (GRADE) framework [52] to rate the certainty of evidence for each outcome. In addition, 2 independent raters (FW and YW) assessed the certainty of evidence using the official GRADE pro GDT software (Evidence Prime Inc), with any disagreements resolved by the corresponding author (RZ). The evidence was rated as very low, low, moderate, or high. Footnotes were provided to explain the reasons for any downgrading of the evidence, which included considerations of risk of bias, inconsistency, indirectness, imprecision, and publication bias.

### Ethical Considerations

This study used literature data and did not require approval from an ethics review committee or patient consent.

## Results

### Search Results

**Figure 1.**
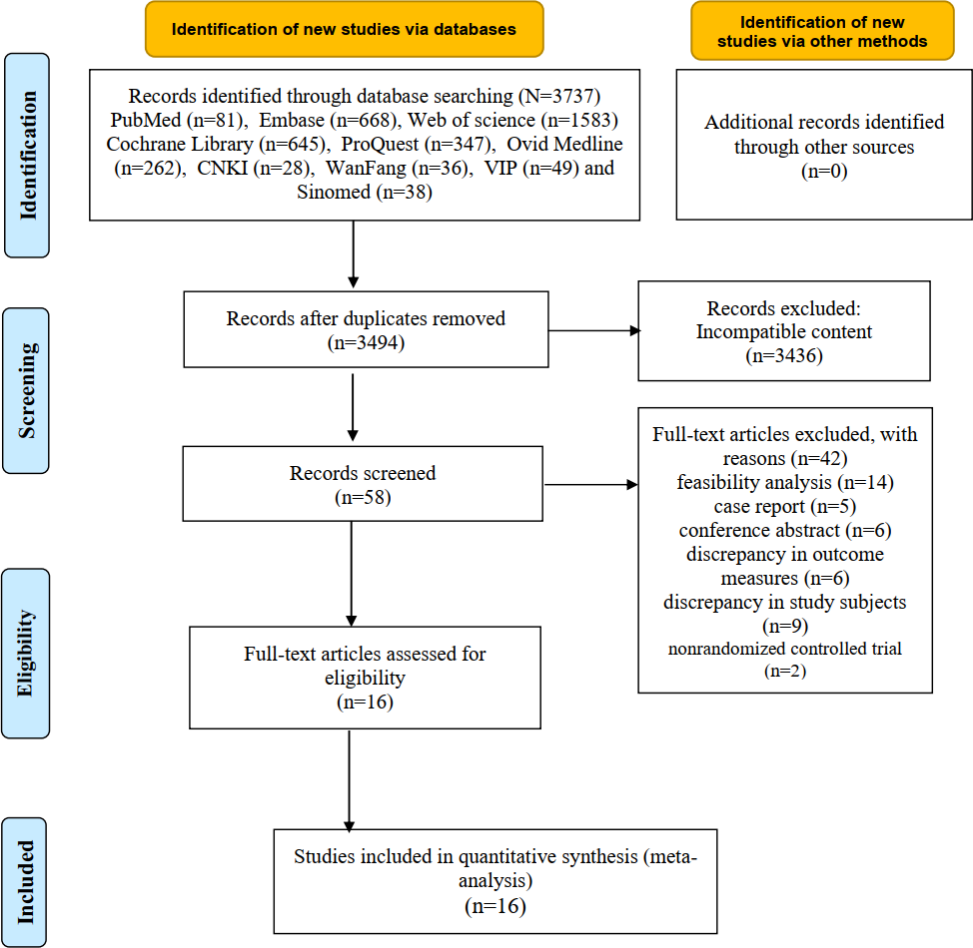
Flowchart of literature screening. CNKI: China National Knowledge Infrastructure; SinoMed: SinoMed Service System; VIP: VIP Database.

A total of 3737 studies were identified from 10 databases. After removing 243 duplicate articles, the titles and abstracts of the studies were initially screened, and 58 full-text manuscripts relevant to this study were identified. Following full-text reading and quality assessment, 16 RCTs [16,17,30,32-35,53-61] were ultimately included for analysis. The specific search and inclusion process is illustrated in Figure 1, and the excluded articles after rescreening, along with the reasons, are detailed in Multimedia Appendix 2.

### Characteristics of the Studies

Multimedia Appendix 3 summarizes the basic characteristics of the included studies. The 16 studies (published between 2020 and 2025) comprised a total of 1356 participants, with sample sizes ranging from 42 to 180. The studies originated from Brazil [53], Korea [30,54], France [17,35], Spain [16], Turkey [34], and China [32,33,55-61]. Patient conditions included cardiovascular diseases [30,35,53,55,60], postcraniotomy [33], postliver transplantation [61], postlaparoscopic sleeve gastrectomy [34], conventional oncologic surgery [32], severe respiratory diseases [58], and postmultiple fracture surgery [56], among others. The mean age of patients was generally between 60 and 70 years. Interventions were initiated within 24 hours after ICU admission in 3 studies [35,53,59], within 6 hours in 2 studies [32,34], and on the day of ICU admission once the patient’s condition was stabilized in the remaining 11 studies [16,17,30,33,54-58,60,61].

One study [16] used nonimmersive VR technology, while 15 studies [17,30,32-35,53-61] used fully immersive VR. The intervention directions included (1) early cognitive rehabilitation (training reaction and executive function through games or tasks) [16,33,55,57], (2) early motor rehabilitation (engaging in virtual activities, such as fruit cutting or cycling combined with motion capture) [53,56,58,59,61], (3) early delirium prevention (providing emotional support and cognitive training for multidimensional prevention) [32], (4) VR relaxation therapy (conducting physical and mental relaxation in natural scenes) [17,34,35], (5) VR meditation (improving sleep and mood by combining mindfulness) [30,54], and (6) VR preoperative visit (structured introduction to the ICU environment, treatment process, and rehabilitation) [60]. The intervention protocols were categorized into (1) single-session short-term intervention (15‐30 min at key clinical time points) [17,30,34,35,60], (2) high-frequency short-term intervention during ICU stay (approximately 20 min per session, 1‐3 times daily, lasting 3‐7 d) [16,32,54,55,57-59], (3) medium- to long-term structured intervention (30‐40 min per session, 5 d per wk, lasting 2 wk to 3 mo) [33,56,61], and (4) flexible individualized protocol (adjusted based on patient tolerance) [53]. The studies involved a total of 24 outcome measures, such as cognitive function, delirium, anxiety, depression, pain, sleep, and quality of life, although the assessment tools and observation windows varied across studies.

### Risk of Bias

**Figure 2.**
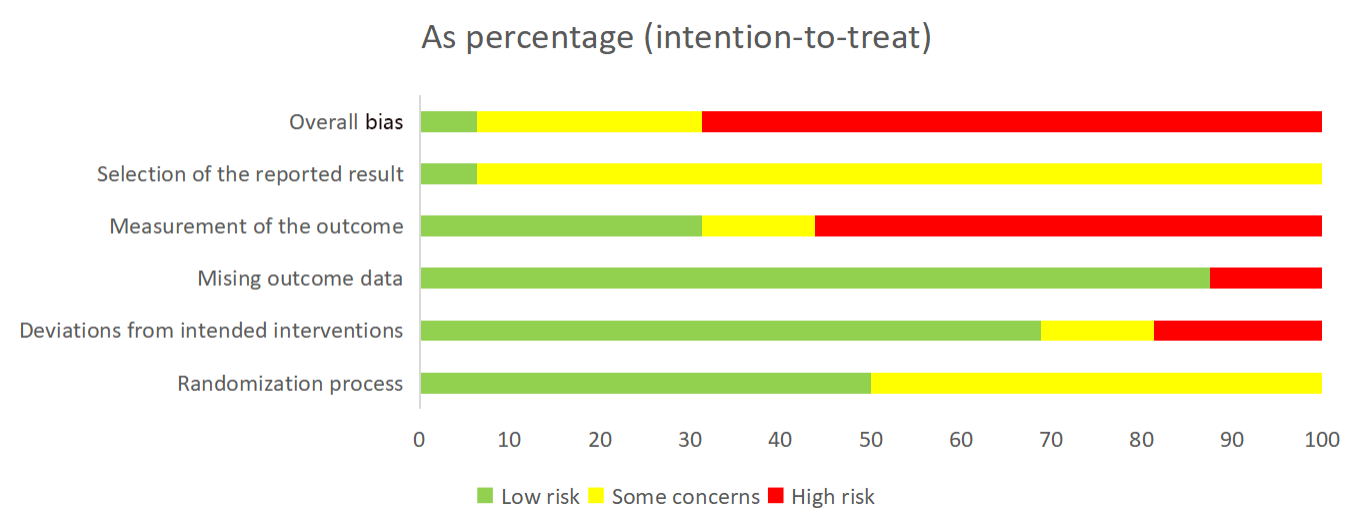
Risk of bias graph for proportion of judgements per item in included studies.

The overall risk of bias for the studies included in this review was high, with summary results presented in Figures2  3, and detailed assessment results available in Multimedia Appendix 4. Furthermore, 1 study [58] was rated as “low,” 5 studies [17,32,34,54,55] raised “some concerns,” and 10 [16,30,33,35,53,56,57,59-61] were rated as “high.” In domain 1 (randomization process), 7 studies [33-35,53,56,60,61] did not describe allocation concealment, and 1 study [32] described neither the method for generating the random sequence nor allocation concealment; all were rated as “some concerns.” For domain 2 (deviations from intended interventions), 4 studies [16,32,55,57] were rated as “some concerns” due to lack of blinding of patients and implementers; 1 study [59] was rated as “high” because it lacked blinding and did not adhere to the intention-to-treat analysis principle. In domain 3 (missing outcome data), 2 studies [16,59] were rated as “high” due to a high rate of loss to follow-up and because the reasons for missing data were potentially related to health status. For domain 4 (measurement of the outcome), 11 studies [16,17,30,32,33,35,53,56,57,60,61] had issues with the lack of blinding of outcome assessors. Among these, 9 studies [16,30,33,35,53,56,57,60,61] were rated as “high” because they measured subjective patient-reported outcomes (eg, pain and anxiety), and 2 studies [17,32] were rated as “some concerns” as their outcome measures were relatively objective. In domain 5 (selection of the reported result), transparency was generally insufficient. Moreover, 4 studies [16,17,35,55] mentioned trial registration but lacked verifiable prespecified analysis descriptions for the reported results, while the remaining 11 studies [30,32-34,53,54,56,57,59-61] did not mention a preregistered protocol; consequently, all were rated as “some concerns.”

**Figure 3.**
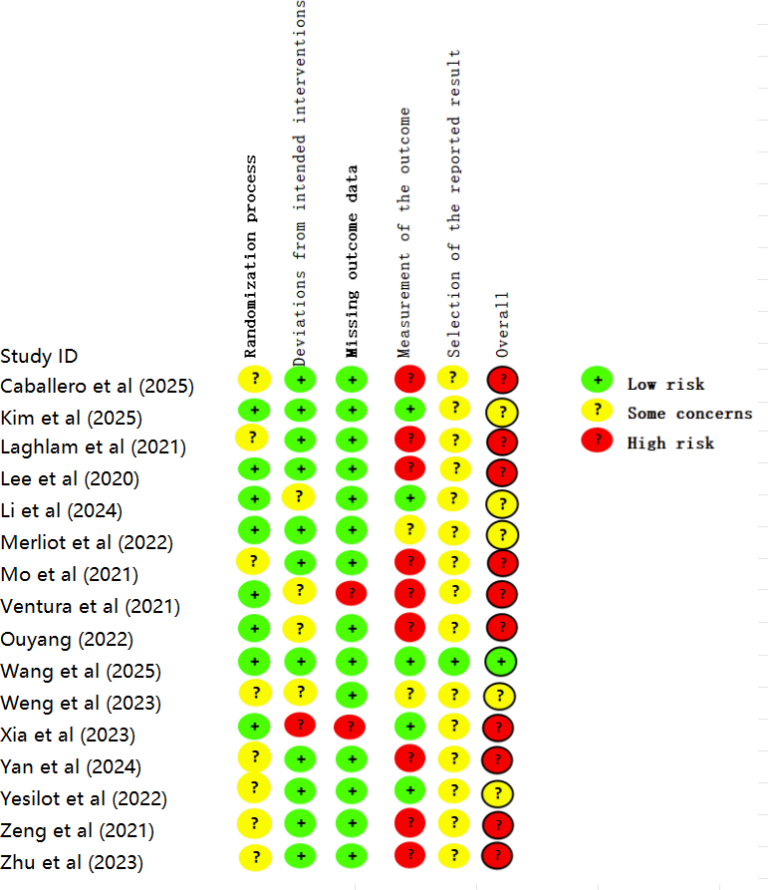
Risk of bias summary Items assessed in each included study [16,17,30,32-35,53-61].

### Clinical Safety and Feasibility

In total, 8 studies [16,17,35,53,55,57-59] reported on safety, 5 studies [16,55,57-59] confirmed the absence of serious adverse events but documented related reactions including dizziness [16,35,57], nausea [35,57], fatigue [16,17], anxiety [16,17], confusion [16,55], visual disturbance [17], headache or discomfort from wearing the headset [55], claustrophobia, agitated withdrawal, and dyspnea due to device displacement [17]. Furthermore, 1 study reported a significant drop in blood pressure during intervention in a patient [59], another reported a case of excessive drowsiness [16], and 1 noted that vital signs in all patients fluctuated within the normal range (±10%) during the intervention [58]. Regarding feasibility, 1 study reported a 78.3% patient recommendation rate and a 90% “good/excellent” experience rating [53]. Meanwhile, 2 studies reported that patients in the VR group demonstrated significantly better outcomes in terms of daily total exercise time, average daily out-of-bed activity time [58], and exercise adherence (with a high adherence rate of 71.74% vs 54.35% at 3 months) [56].

### Results of the Meta-Analysis

Among the included trials, due to the limited number of studies, we conducted meta-analyses on outcome indicators with at least 2 studies. The specific analysis results are as follows.

#### Anxiety

A total of 8 studies (n=801) included anxiety as an outcome measure. According to the predefined data pooling criteria, for the study by Li et al [55], we only pooled the data assessed during ICU treatment using the State-Trait Anxiety Inventory scale. For the study by Merliot-Gailhoustet et al [17], we only pooled the data from the group receiving the DEEPSEN VR system (Saint-Didier-au-Mont-d’Or, France). All studies were analyzed using change-from-baseline values for the pooling.

The meta-analysis revealed that, in terms of the average effect, VR-ERI did not show a statistically significant effect on reducing patient anxiety (SMD −0.86, 95% CI −1.85 to 0.13). Given that higher scores on all assessment tools indicated greater anxiety, the negative point estimate suggests that VR-ERI may reduce anxiety. However, the 95% PI was wide and included the null value (−3.75 to 2.03), indicating considerable uncertainty about the intervention’s effect in future settings, ranging from a substantial benefit to potential harm. Therefore, these findings require validation in further studies.

**Figure 4.**
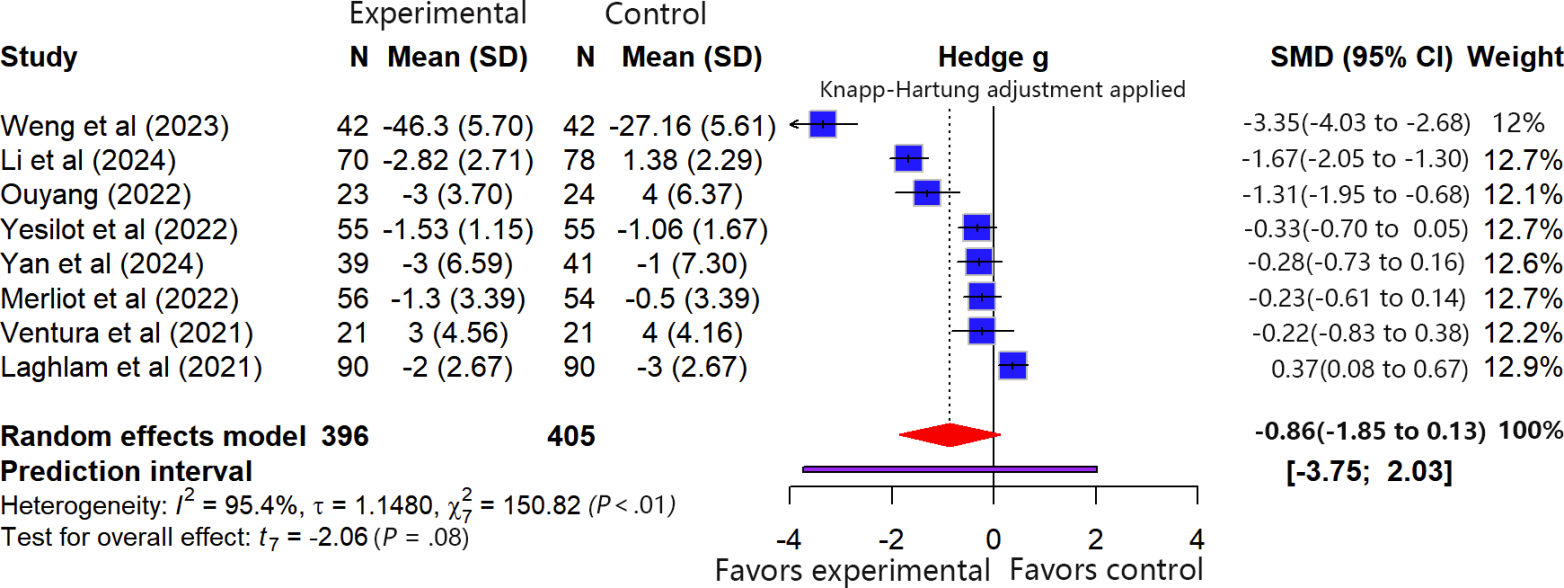
Forest plot of the meta-analysis for anxiety outcomes [16,17,32,34,35,55,57,60].

Extremely high heterogeneity was detected among the studies (*I*²=95.4%, *τ*=1.1480, *τ*²=1.3179; Cochran Q=150.82, df=7; *P*<.01; Figure 4). Exploratory subgroup analyses were performed based on VR intervention content, timing, patient population, and type of anxiety assessment tool, but no potential source of heterogeneity was identified. Sensitivity analysis (leave-one-out method) indicated that the pooled effect size was sensitive to the removal of individual studies, with a maximum change of 0.3478, exceeding the prespecified threshold of 0.1, suggesting that the results may not be robust. The study by Weng et al [32] had the most substantial influence on the overall effect estimate. After excluding this study, the pooled effect size remained statistically nonsignificant (SMD −0.51, 95% CI −1.17 to 0.15; 95% PI −2.31 to 1.28), and heterogeneity remained high (*I*²=92.5%, *τ*=0.6806, *τ*²=0.4632; Cochran Q=132.89, df=6; *P*<.01), further supporting the instability of the findings.

According to the GRADE framework, the certainty of evidence for anxiety was rated as “very low.” The downgrading was based on (1) serious risk of bias in the included studies (4 studies [16,35,57,60] with high risk), (2) very high heterogeneity among studies, (3) imprecision of the pooled effect estimate (the 95% CI spanned both the line of null effect and the threshold for clinically meaningful benefit), and (4) potential publication bias. Although the pooled effect size suggested a potential moderate-to-large effect, an upgrade was not considered after weighing the above limitations (detailed assessment is available in Multimedia Appendix 5).

#### Subjective Sleep Quality (Scale Assessment)

In total, 6 studies [30,32,54,55,57,60] (n=500) assessed subjective sleep quality during ICU stay. In accordance with predefined pooling criteria, we extracted data from the second intervention day for Kim and Kang [54] and the Richards-Campbell Sleep Questionnaire (RCSQ)–measured sleep data only for Ouyang [57]. While all studies reported comparable baseline characteristics, specific data were provided by only 3 [32,55,60]. To maximize data utility, the meta-analysis was performed using postintervention outcomes.

**Figure 5.**
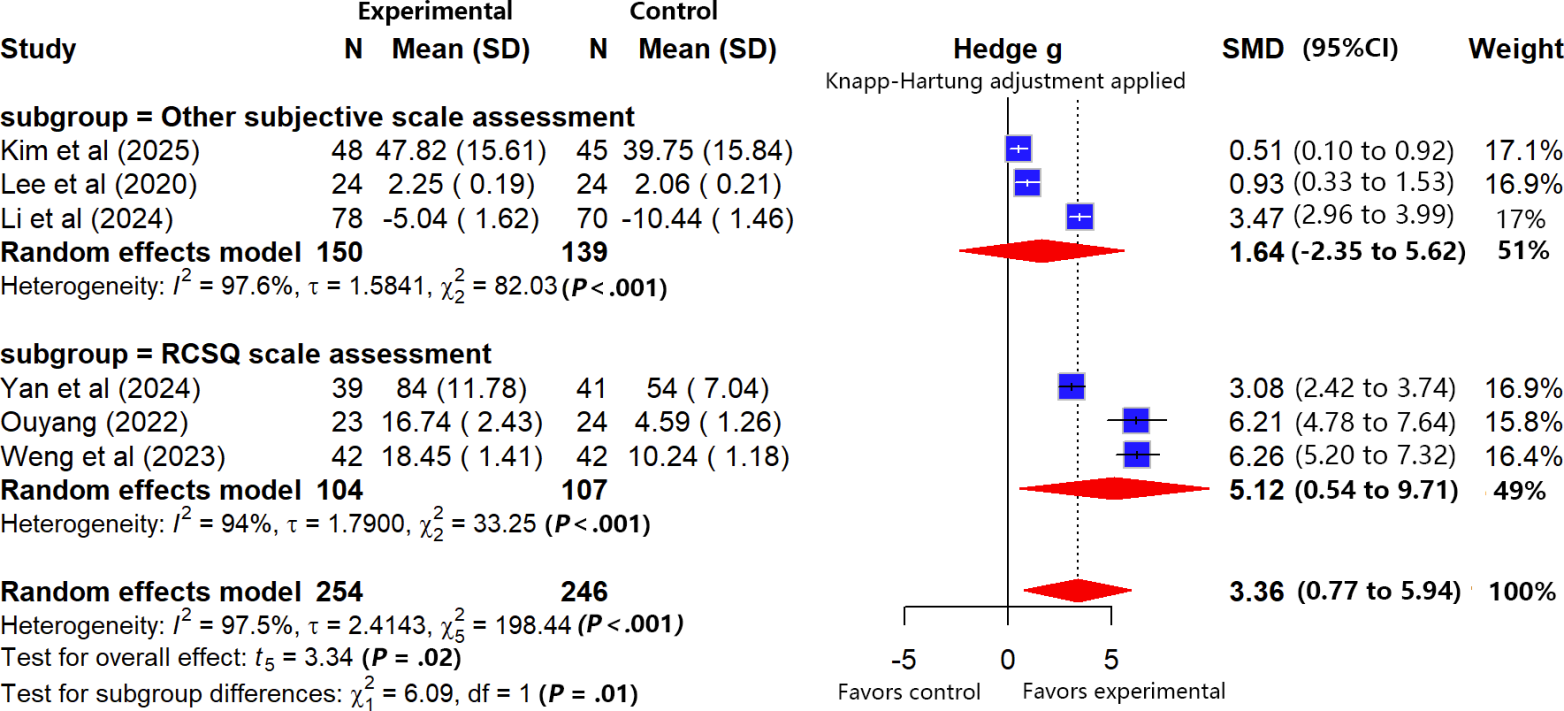
Forest plot of the subgroup analysis for subjective sleep quality [30,32,54,55,57,60].

Meta-analysis revealed considerable heterogeneity (*I*²=97.5%, *τ*=2.4143, *τ*²=5.8288; Cochran Q=198.44, df=5; *P*<.01), likely due to differences in populations, interventions, and assessments. Sensitivity analysis (leave-one-out method) confirmed the robustness of the results, as the maximum change in effect size after excluding any single study was 0.0347 (below the prespecified 0.1 threshold), with no specific source of heterogeneity identified. The pooled results (Figure 5) indicated a significant positive effect of VR-ERI on improving patients’ subjective sleep quality compared with usual care (SMD 3.36, 95% CI 0.77-5.94). For consistent interpretation, Pittsburgh Sleep Quality Index scores were reversed so that higher scores uniformly indicated better sleep quality across all scales; thus, the positive SMD reflects an enhancing effect of VR-ERI, although more studies are needed to confirm the precision of this estimate.

Subgroup analysis was conducted after unifying scale directions to assess how the inherent characteristics of different instruments might influence the pooled result. The analysis showed that VR-ERI did not significantly improve sleep when assessed with generic scales (SMD 1.64, 95% CI −2.35 to 5.61). In contrast, a positive effect was observed with the ICU-specific RCSQ (SMD 5.12, 95% CI 0.54 to 9.71). The between-subgroup difference was statistically significant (*χ*²_1_=6.1; *P*=.014), indicating that the type of assessment tool is a significant effect modifier, with the RCSQ potentially being more sensitive to VR-ERI–related sleep improvements in the ICU setting.

According to the GRADE approach, the certainty of evidence for subjective sleep quality during ICU treatment was rated as “very low.” The reasons for downgrading included (1) a serious risk of bias in the included studies (3 studies [30,57,60] with high risk), (2) very high heterogeneity among studies, and (3) extreme imprecision of the pooled effect estimate (the 95% CI was extremely wide, spanning the range from moderate to very large effects). Considering all the downgrading factors above, an upgrade was not applied despite the large pooled effect size (for detailed assessment, refer to Multimedia Appendix 5).

#### Objective Sleep Quality (Smart Wristband Monitoring)

In total, 2 studies [30,54] (n=141) monitored objective sleep quality using smart wristbands. Key metrics included total sleep time (TST), defined as the actual duration of sleep from onset to awakening; wake after sleep onset (WASO), representing total wake time during the sleep period as an indicator of sleep fragmentation; and sleep efficiency, calculated as the percentage of TST relative to total time in bed, with higher values indicating better sleep quality. A pooled analysis was performed using change-from-baseline values, with specific results as follows.

#### TST

**Figure 6.**
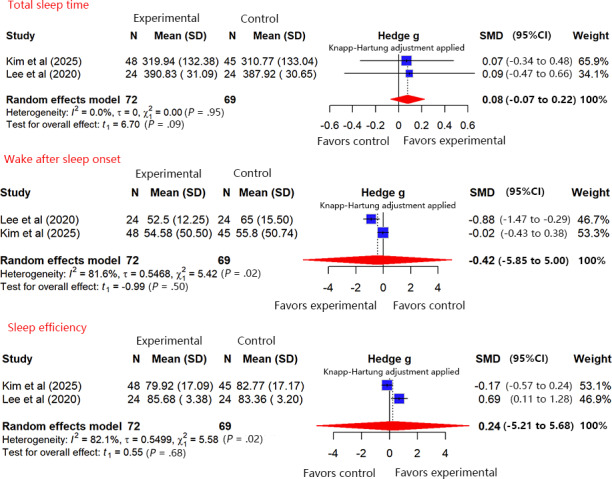
Forest plot of the pooled analysis for objective sleep quality [30,54].

The meta-analysis indicated no heterogeneity among the studies (*I*²=0%, *τ*=0, *τ*²=0; Cochran Q=0, df=1; *P*=.95). Sensitivity analysis (leave-one-out method) indicated that the results were robust. The maximum change in the effect size after excluding any single study was 0.0375, which is below the prespecified threshold of 0.1. The meta-analysis (Figure 6) showed that, regarding the average effect, VR-ERI did not lead to a statistically significant improvement in patients’ TST compared with usual care (SMD 0.08, 95% CI −0.07 to 0.22). The positive SMD suggests that the direction of the effect may favor VR-ERI, indicating an increase in TST. According to the GRADE approach, the certainty of evidence for objective TST during ICU treatment was rated as “moderate.” The rating was downgraded due to a high risk of bias in 1 study [30]. No downgrading was applied for other domains, as there was no heterogeneity among studies and the pooled effect estimate was relatively precise (for detailed assessment, refer to Multimedia Appendix 5).

#### WASO

The meta-analysis indicated substantial heterogeneity among the studies (*I*²=81.6%, *τ*=0.5468, *τ*²=0.2990; Cochran Q=5.42, df=1; *P*=.02). Sensitivity analysis showed that the results were robust (the maximum change in the effect size after excluding any single study was 0.0397, below the threshold of 0.1). The meta-analysis (Figure 6) showed that, regarding the average effect, VR-ERI did not lead to a statistically significant reduction in WASO compared with usual care (SMD −0.42, 95% CI −5.85 to 5.00). The negative SMD indicates that the direction of the effect points toward a reduction in WASO, signifying an improvement in sleep maintenance. According to the GRADE approach, the certainty of evidence for objective WASO during ICU treatment was rated as “very low.” The rating was downgraded due to (1) a very limited number of included studies with risk of bias, (2) high heterogeneity among studies, and (3) extreme imprecision of the pooled effect estimate (the 95% CI was exceptionally wide; for detailed assessment, refer to Multimedia Appendix 5).

#### Sleep Efficiency

The meta-analysis indicated substantial heterogeneity among the studies (*I*²=82.1%, *τ*=0.5499, *τ*²=0.3024; Cochran Q=5.58, df=1; *P*=.02). Sensitivity analysis indicated robust results (the maximum change in the effect size after excluding any single study was 0.0432, below the threshold of 0.1). Regarding the average effect, the meta-analysis (Figure 6) showed that VR-ERI did not lead to a statistically significant improvement in sleep efficiency compared with usual care (SMD 0.24, 95% CI −5.21 to 5.68). The positive SMD indicates that the direction of the effect points toward an improvement in sleep efficiency. According to the GRADE approach, the certainty of evidence for objective sleep efficiency during ICU treatment was rated as “very low.” The rating was downgraded due to (1) risk of bias in the included studies, (2) substantial heterogeneity among studies, and (3) extreme imprecision of the pooled effect estimate (refer to Multimedia Appendix 5 for detailed assessment).

#### Pain

In total, 3 studies included pain level during ICU treatment as an outcome measure. According to the predefined data pooling criteria, for the study by Merliot-Gailhoustet et al [17], we only included the pain data from patients who received the DEEPSEN VR system. The pain outcome measure was analyzed using change-from-baseline values for the pooled analysis.

**Figure 7.**
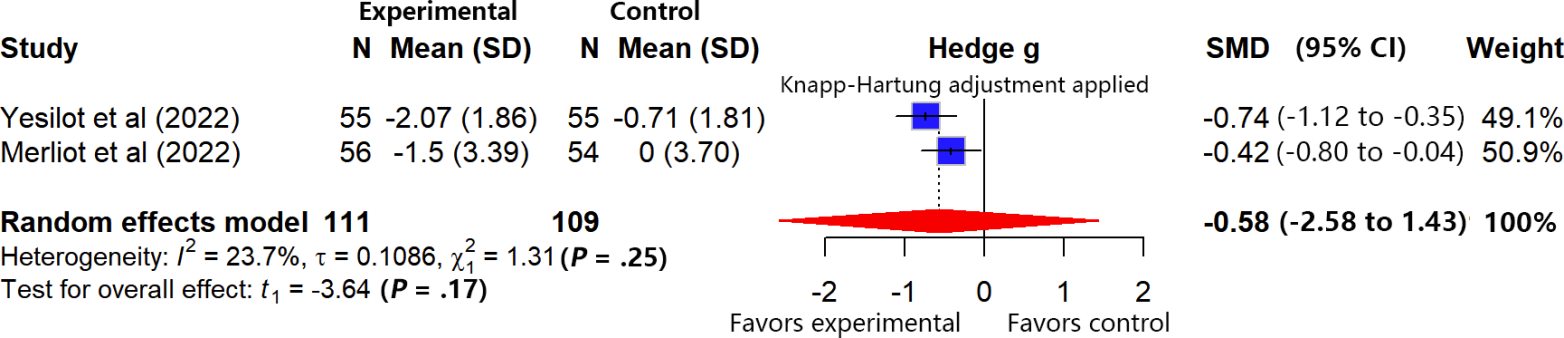
Forest plot for pain outcomes during the intensive care unit stay [17,34].

Initial meta-analysis revealed extremely high heterogeneity among the studies (*I*²=95.1%, *τ*=0.7550, *τ*²=0.5700; Cochran Q=41.10, df=2; *P*<.01). Regarding the average effect, VR-ERI did not show a statistically significant difference in reducing patients’ ICU-related pain compared with usual care (SMD −0.13, 95% CI −2.06 to 1.79). Sensitivity analysis (leave-one-out method) indicated that the results may not be robust. The maximum change in the effect size after excluding a single study was 0.4062, which exceeds the prespecified threshold of 0.1. The study with the largest influence was by Laghlam et al [35]. Upon verification, this study was a noninferiority trial comparing VR therapy with another analgesic intervention. Pooling its control group with standard usual care controls could confound the pain outcome. Therefore, the study by Laghlam et al [35] was excluded, and the pooled result from the remaining 2 studies [17,34] (n=220) was used as the primary evidence for assessing the effect of VR-ERI on pain during ICU treatment. The meta-analysis of the 2 remaining studies showed low heterogeneity (*I*²=23.7%, *τ*=0.1086, *τ*²=0.0118; Cochran Q=1.31, df=1; *P*=.25; Figure 7). Regarding the average effect, VR-ERI did not demonstrate a statistically significant effect on reducing pain during ICU treatment compared with usual care (SMD −0.58, 95% CI −2.58 to 1.43). Subgroup analysis was not performed due to the limited number of studies.

According to the GRADE criteria, the certainty of evidence for the outcome of pain during ICU treatment was rated as “low.” The reasons for downgrading include (1) a high risk of bias in the included primary studies and (2) serious imprecision, as the 95% CI of the pooled effect is extremely wide and includes the line of null effect. As the heterogeneity among the remaining studies was low after the exclusion of 1 study based on clinical heterogeneity, no downgrading was applied for inconsistency (refer to Multimedia Appendix 5 for detailed assessment).

#### Balance

**Figure 8.**
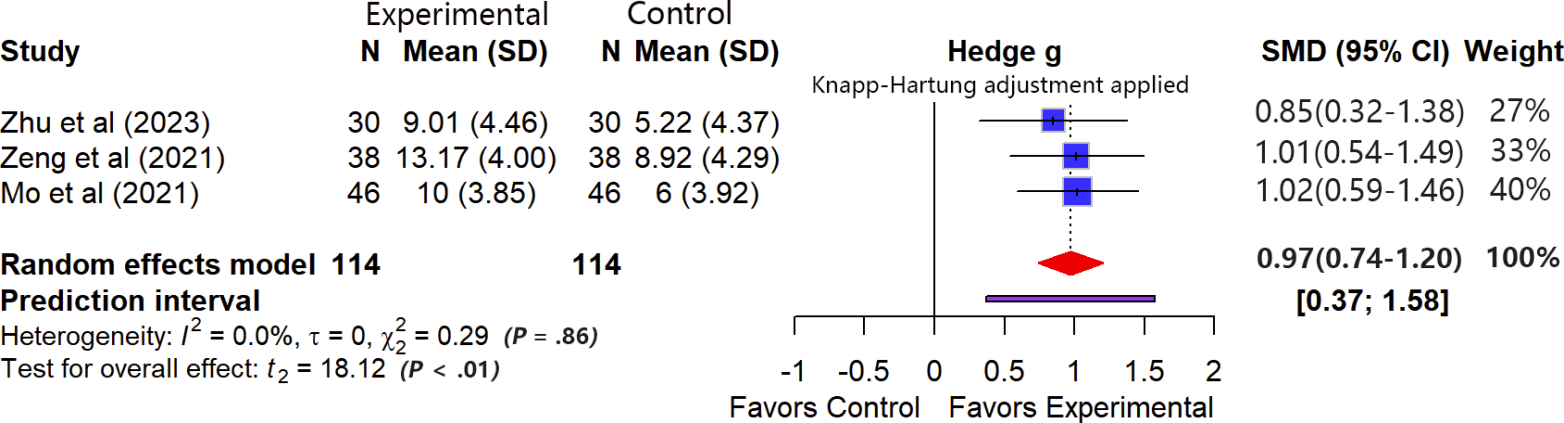
Forest plot for balance outcomes [33,56,61].

In total, 3 studies [33,56,61] (n=228) assessed patients’ balance function using the Berg Balance Scale at short-term follow-up. We pooled change-from-baseline values for analysis. No heterogeneity was observed among the included studies (*I*²=0%, *τ*=0, *τ*²=0; Cochran Q=0.29, df=2; *P*=.86). The meta-analysis showed that, in terms of the average effect, VR-ERI improved patients’ balance function during short-term follow-up after discharge compared with usual care (SMD 0.97, 95% CI 0.74 to 1.20). Furthermore, the 95% PI (0.37-1.58) lies entirely to the right of the line of null effect, suggesting that the positive effect of VR-ERI on balance function is likely to remain at a clinically meaningful level in most future, similar studies (Figure 8). Sensitivity analysis (leave-one-out method) indicated that the results were robust. The maximum change in the effect size after excluding any single study was 0.0461, which is below the prespecified threshold of 0.1. Subgroup analysis was not performed due to the limited number of studies.

According to the GRADE approach, the certainty of evidence for balance function at short-term follow-up was rated as “moderate.” The rating was downgraded because all included primary studies were assessed as having a high risk of bias. No downgrading was applied for inconsistency or imprecision, as no heterogeneity was observed among studies, and the pooled effect estimate was precise. Although the pooled effect size was large and clinically meaningful, an upgrade was not performed. This decision was made to maintain a cautious interpretation, considering that the methodological limitations commonly present in the existing studies could affect the validity of the effect estimate (refer to Multimedia Appendix 5 for the detailed evaluation process).

#### Cognitive Function

A total of 3 studies [33,55,57] (n=271) were included that assessed cognitive function at short-term follow-up. According to the predefined data pooling criteria, for the study by Zeng [33], we only included the results obtained using the Montreal Cognitive Assessment (MoCA) scale. All analyses were based on change-from-baseline values.

**Figure 9.**
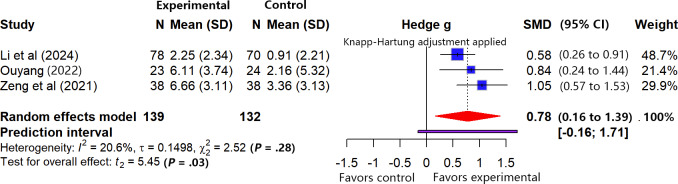
Forest plot of the meta-analysis for cognitive function outcomes [33,55,57].

The meta-analysis (Figure 9) indicated mild heterogeneity among the studies (*I*²=20.6%, *τ*=0.1498, *τ*²=0.0224; Cochran Q=2.52, df=2; *P*=.28). Sensitivity analysis (leave-one-out method) showed that the results are robust, as the maximum change in the effect size after excluding any single study was 0.0218, which is below the threshold of 0.1. The pooled results indicated that, on average, VR-ERI showed a statistically significant trend toward improving patients’ cognitive function compared with usual care (SMD 0.78, 95% CI 0.16 to 1.39). However, the 95% PI (−0.16 to 1.71) crossed the line of null effect, suggesting that the beneficial effect of VR-ERI on cognitive function in most future, similar studies is unstable—it could be beneficial or show no difference and therefore requires further exploration in future research. Subgroup analysis was not performed due to the limited number of included studies.

According to the GRADE framework, the quality of evidence was rated as “low.” This rating was based on (1) all 3 included studies [33,55,57] were rated as having a high risk of bias, and (2) imprecision of the pooled effect estimate. Although the pooled effect size suggests a moderate effect, the methodological flaws in the studies significantly reduce our confidence in this estimate. Specific rating details are provided in Multimedia Appendix 5.

#### Limb Motor Function

In total, 2 studies [33,56] (n=168) assessed the improvement of patients’ limb motor function during short-term follow-up using the Fugl-Meyer Assessment. As the study by Zeng [33] reported upper limb and lower limb function scores separately, we applied the weighted mean method to combine the function scores from different body parts within the same study into an overall limb motor function score. All analyses were based on change-from-baseline values.

**Figure 10.**
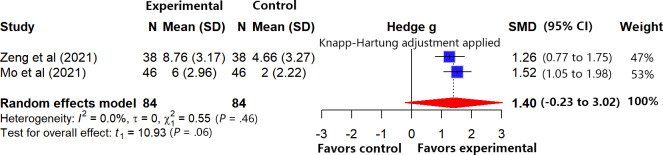
Forest plot for limb motor function [33,56].

No heterogeneity was observed between the studies (*I*²=0%, *τ*=0, *τ*²=0; Cochran Q=0.55, df=1; *P*=.46). The meta-analysis (Figure 10) indicated that, based on the average effect, VR-ERI showed a trend toward improving patients’ limb motor function at short-term follow-up after discharge compared to usual care (SMD 1.40, 95% CI −0.23 to 3.02), although the difference was not statistically significant and requires further verification. According to the GRADE framework, the quality of evidence was rated as “low.” This rating was based on (1) both included studies had a high risk of bias, and (2) the pooled effect estimate was extremely imprecise. Although the pooled effect size suggests a potentially large effect, we did not upgrade the rating due to the very limited evidence base and the low quality of the studies (Multimedia Appendix 5).

### Descriptive Integration Results

#### Overview

We performed descriptive analysis on outcomes with fewer than 2 included studies and those unsuitable for meta-analysis due to differences in assessment methods or data collection time points. Given the limited number of studies, further validation of the intervention effects on these outcomes is currently lacking, and more high-quality RCTs are needed in the future to evaluate them.

#### Motor Function Outcomes (Muscle Strength, Grip Strength, Patient Engagement, and Functional Outcomes)

VR-ERI demonstrated multifaceted benefits on motor function, including improved muscle strength. In total, 2 studies supported this finding; Zhu et al [61] reported significantly higher Manual Muscle Testing scores in the VR-ERI group versus controls (*t*_58_=2.33, *P*=.02), while Wang et al [58] observed stronger muscle strength via the Medical Research Council scale (*F*_1,66_=7.9, *P*=.006), with a maintained intervention effect over time (*F*_7,66_=50.1, *P*<.001). Due to insufficient raw data, a meta-analysis was not conducted, and further studies are needed to confirm these results. In contrast, findings regarding grip strength improvement were inconsistent. Both Xia et al [59] and Wang et al [58], using a dynamometer, reported no statistically significant between-group difference in absolute grip strength post intervention. Notably, Wang et al [58] observed significant improvement over time in both groups and a significant group-time interaction, suggesting VR-ERI may influence the recovery trajectory. However, its advantage over conventional rehabilitation requires further validation.

Furthermore, VR-ERI also enhanced patient engagement. Both Xia et al [59] and Wang et al [58] reported that the intervention group had significantly longer daily exercise duration than the control group. Mo et al [56] further supported this, finding a higher proportion of patients with high exercise compliance in the VR-ERI group (33/46, 71.74%) versus the control group (25/46, 54.35%) via a self-developed questionnaire. This may be attributed to the inherent enjoyment and real-time feedback of VR-based training, although limited raw data necessitates further research. Regarding functional outcomes, VR-ERI also demonstrated positive effects. Mo et al [56] observed a higher proportion of patients attaining the highest walking ability level (Functional Ambulation Category grade 5) in the intervention group (22/46, 47.93%) versus the control group (14/46, 30.43%). Meanwhile, Xia et al [59] reported a significant reduction in ICU-acquired weakness incidence with VR-ERI (intervention: 13.5% vs control: 35.5%). Future high-quality studies are needed to confirm these effects.

#### Psychological Function Outcomes (Depression, Posttraumatic Stress Disorder, Somatic Symptoms, and Perceived Well-Being)

The effects of VR-ERI on psychological function were inconsistent. Navarra-Ventura et al [16] found no significant between-group differences in depression or posttraumatic stress disorder at 1 and 12 months post-ICU discharge. In contrast, Ouyang [57] reported a significant reduction in depression during ICU treatment. However, due to differing assessment timepoints between the 2 studies, meta-analysis was not performed, and these divergent conclusions require further validation. Li et al [55] observed better improvement in somatic symptoms and higher perceived well-being in the intervention group at 1 week during ICU stay, but these differences were not maintained 1 month after discharge. Due to differing assessment timepoints, meta-analysis was not performed, and the long-term psychological effects of VR-ERI require further investigation.

#### Cognitive Function Outcomes (Delirium)

Kim and Kang [54] used the Confusion Assessment Method (CAM) for ICU scale to evaluate the effect of VR-ERI on delirium incidence and found no significant difference between the 2 groups (12.2% vs 12.8%, *χ*²_1_=0.01; *P*=.94). In contrast, Weng et al [32] used the CAM scale to assess delirium severity and reported a statistically significant difference in CAM scores between groups (mean 2.25, SD 0.44 vs mean 6.37, SD 1.05, *t*_82_=23.453; *P*<.05). Future high-quality studies with standardized assessment criteria are recommended to validate the intervention effects.

#### ICU Length of Stay and Recovery Quality

Regarding ICU length of stay, both Xia et al [59] and Wang et al [58] reported significantly shorter stays for the VR-ERI group versus controls, suggesting faster patient turnover. However, a meta-analysis was not feasible due to incomplete raw data. For quality of recovery, Zhu et al [61] (using the Postoperative Quality Recovery Scale) found significantly better recovery rates in the VR-ERI group for physiological function and activity domains. Li et al [55] noted improved mental health scores (12-Item Short Form Health Survey Mental Component Score) at 1 week post intervention, though physical health scores (12-Item Short Form Health Survey Physical Component Score) showed no difference, and no between-group differences remained significant at the 3-month follow-up.

### Small-Study Effects

Although the number of included studies for all outcomes in this review did not meet the recommended threshold for conventional statistical tests of publication bias (≥10 studies), we still performed an exploratory analysis on the anxiety outcome, which had the largest number of included studies (n=8). This was done to preliminarily assess the potential impact of bias on the robustness of the conclusions.

**Figure 11.**
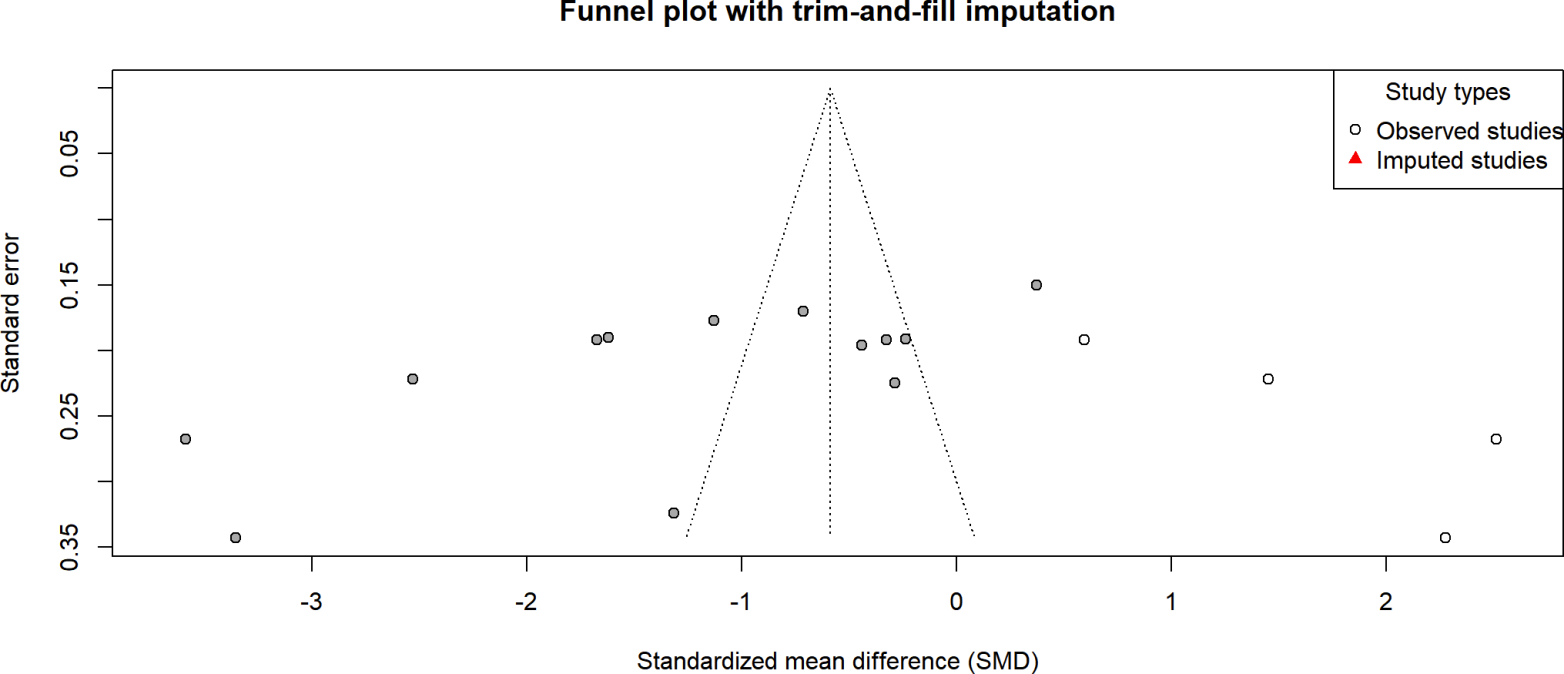
Funnel plot for anxiety outcomes[16,17,32,34,35,55,57,60].

The funnel plot (Figure 11) appeared approximately symmetrical by visual inspection. Statistical test results showed that the Egger linear regression test did not indicate significant small-study effects (*t*_6_=−1.94; *P*=.10). However, the Begg rank correlation test suggested the possible presence of publication bias (*z*=−2.47, *P*=.01). To quantify the potential impact of missing studies on the pooled result, a trim-and-fill analysis was conducted. The analysis estimated that 1 potentially missing study needed to be imputed. The adjusted pooled effect size changed from the original SMD −0.862 (95% CI −1.853 to 0.129) to SMD −0.495 (95% CI −1.719 to 0.729), a change of 0.367. The decrease in the absolute value suggests that, if bias exists, its influence may have led to an overestimation of the intervention effect.

We fully acknowledge that with only 8 included studies [16,17,32,34,35,55,57,60] and substantial heterogeneity present, the statistical power of these tests is limited. Furthermore, heterogeneity itself can interfere with the interpretation of funnel plot asymmetry. Therefore, the above results should be considered exploratory. In summary, the current analysis, while suggesting that publication bias cannot be entirely ruled out, does not provide evidence from the Egger test supporting significant small-study effects. Moreover, the CI for the effect size adjusted by the trim-and-fill method remained wide and crossed the line of null effect. This indicates that, even if potential bias exists, the current conclusion regarding the anxiety outcome is stable. However, it serves as a reminder to interpret the magnitude of this effect size with caution in the future.

## Discussion

### Principal Results

This review systematically assessed the feasibility, safety, and rehabilitation outcomes of VR-ERI in critically ill patients across 16 RCTs [16,17,30,32-35,53-61] (n=1356). VR-ERI was well-accepted and associated with high exercise adherence. Vital signs remained within acceptable ranges during use, and no serious adverse events were reported; however, mild dizziness, nausea, and fatigue occurred in some studies, underscoring the need for standardized safety monitoring. Regarding efficacy, VR-ERI improved balance during short-term follow-up, supported by low heterogeneity, narrow PIs, and moderate-certainty evidence, indicating robust and potentially generalizable benefit. VR-ERI also shows potential to improve in-ICU anxiety and subjective sleep quality, as well as short-term cognitive and motor function, particularly sleep, when assessed with the RCSQ. However, it did not significantly improve objective sleep parameters or in-ICU pain. For most outcomes, the certainty of evidence was low or very low due to implementation bias (eg, lack of blinding), study inconsistency, and imprecision in pooled estimates. Thus, results should be interpreted cautiously, and current evidence remains insufficient for definitive conclusions. Some outcomes could not be pooled due to limited studies. Further high-quality trials are needed to confirm these findings and clarify the clinical value of VR-ERI in critical care.

VR-ERI may reduce anxiety in critically ill patients, a finding consistent with recent reviews in both critical care [36] and elective surgery [62], suggesting VR could broadly mitigate hospital-related anxiety. Stress recovery theory [63] offers a possible explanation, proposing that exposure to natural landscapes can activate the parasympathetic nervous system and promote emotional relaxation. Research confirms that biophilic elements aid stress recovery [64]. Most VR-ERI scenarios in the included studies incorporated natural views (eg, mountains and rivers), which may help block typical ICU stressors, such as constant light, noise, and isolation. However, the pooled effect in this analysis was not statistically significant, and the 95% PI crossed zero, indicating instability in the estimate. The included studies also had a high risk of bias, with substantial inconsistency and imprecision, yielding very low certainty of evidence. Thus, future rigorous RCTs with adequate sample sizes and transparent reporting are needed to clarify VR’s effect on anxiety in this population.

VR-ERI shows potential in improving ICU patients’ subjective sleep quality, likely due to its intervention design—2 protocols [30,54] used VR meditation and hypnosis modules to promote relaxation and distraction, enhancing perceived sleep quality [30,54]. However, objective parameters measured with smart wristbands (eg, TST and sleep efficiency) did not improve concurrently, suggesting VR may modulate sleep perception rather than alter sleep physiology. A recent network meta-analysis [65] found that simple interventions like eye masks and earplugs offer more consistent sleep benefits in critically ill patients, while the effect of VR meditation remains unestablished, underscoring VR’s uncertain role in sleep intervention. Subgroup analysis indicated positive effects only when sleep was assessed with the RCSQ, not with other subjective scales. This highlights how tool selection influences outcomes, possibly because the RCSQ focuses more on immediate sleep depth and is designed specifically for critically ill patients. Future research should use standardized, population-specific sleep assessment tools to accurately capture intervention effects. Current evidence is limited by low certainty. Future studies should develop standardized VR sleep protocols, clarify optimal dosage, and incorporate objective measures, such as polysomnography or validated wearable monitoring, to systematically evaluate VR’s impact on sleep architecture, thereby building a more reliable evidence base for practice.

We also observed that VR-ERI can improve balance (measured by Berg Balance Scale) and limb motor function (measured by Fugl-Meyer Assessment) in critically ill patients during short-term follow-up. This finding is consistent with the review by He et al [37] on VR for early motor intervention in this population. By providing an immersive environment, VR compensates for the sensory and interaction deficits resulting from prolonged immobilization and isolation. It enhances sensorimotor pathways and balance-related neuroplasticity through visual-vestibular-proprioceptive integration [66]. Furthermore, VR-ERI engages both cognitive and motor functions simultaneously [67] and promotes cortical activation via feedback-driven learning and task-specific adaptation [68,69]. The engaging and gamified design of VR-ERI may also improve patient adherence to exercise, which could explain its advantage over traditional motor rehabilitation. Improvement in balance is supported by moderate-quality evidence and relatively narrow PIs, suggesting that standardized VR-ERI motor activities could be implemented early in critical care rehabilitation, with attention to personalized parameters and long-term effect tracking. However, evidence for limb motor improvement remains low in certainty and requires further rigorous validation.

However, VR-ERI did not demonstrate a statistically significant effect on reducing pain in critically ill patients during ICU treatment, a result that may be associated with several factors. The pain experience of critically ill patients is particularly complex, as frequent threats to psychological and physical integrity make their pain difficult to manage effectively [70]. Neuroimaging studies have shown that receiving VR intervention during painful stimuli can significantly reduce excitability in brain regions associated with pain processing, with a higher level of VR immersion correlating with stronger inhibitory effects [71]. Concurrently, immersive VR devices can divert attention away from nociceptive stimuli by isolating patients’ audiovisual connection to the external environment [72]. Nevertheless, in this study, the reported pain scores were relatively low in both groups, which might be related to insufficient immersion of the VR experience, inadequate intervention dosage, or the widespread use of analgesic medications. This warrants further investigation in future studies.

VR-ERI shows a potential trend for improving cognitive function in short-term follow-up, although the 95% PI crosses the null effect line, indicating unstable results. This may stem from several factors. First, the pathophysiology of post-ICU cognitive impairment is complex (eg, neuroinflammation and blood-brain barrier disruption) [73]; for instance, underlying neural damage in conditions like posterior cortical atrophy may limit response to conventional interventions [74], highlighting the need for more targeted programs. Second, widely used screening tools, such as the Mini-Mental State Examination and MoCA, may lack sensitivity to detect subtle, clinically meaningful improvements in critically ill patients. The Mini-Mental State Examination, although brief and simple, has a ceiling effect and inadequately assesses executive function and complex attention [75]. The MoCA is more sensitive to mild impairment but remains a broad screening tool that may miss specific post-ICU executive deficits (eg, inhibitory control and cognitive flexibility) [76]. Therefore, future studies should develop VR modules tailored to ICU-related cognitive impairment and consider longer intervention duration. A multilevel assessment strategy is recommended, combining screening scales with computerized neuropsychological tests (eg, flanker tasks) for objective, quantitative data [77]. Integrating VR with other rehabilitation methods into a multidimensional intervention may also help achieve meaningful cognitive gains. Given the low certainty of current evidence, these findings should be interpreted with caution.

This systematic review is the first to focus on VR intervention studies conducted during the early ICU rehabilitation phase. By systematically searching ten Chinese and English databases and implementing independent dual-reviewer screening and data extraction, it strictly adhered to the PRISMA 2020 guidelines, ensuring a transparent process and reproducible results. The review comprehensively used the Cochrane RoB 2.0 tool for risk of bias assessment and the GRADE framework for evidence grading, supplemented by sensitivity analyses to verify the robustness of the findings. It summarizes and analyzes the safety and clinical feasibility of VR intervention in critically ill patients, while systematically elucidating the efficacy profile of VR on relevant outcomes during both the ICU stay and at short-term follow-up. This provides an important reference for advancing the translational application of VR technology in this patient population.

### Limitations and Future Recommendations

Several limitations should be acknowledged. First, the literature search was restricted to Chinese and English publications and excluded trial registries and gray literature, potentially introducing omission and language bias. Second, the limited number of studies and small total sample size reduce statistical power, which may affect the robustness and generalizability of the pooled results. Third, substantial clinical and methodological heterogeneity existed across studies, including variations in VR immersion levels, protocol design, dosage, and outcome measures, contributing to high statistical heterogeneity. Inadequate reporting of precise dosage parameters also limited dose-response analyses. Fourth, most included studies had a high risk of bias due to issues such as unclear allocation concealment, lack of assessor blinding, and inherent challenges in blinding participants and personnel. Consequently, the certainty of evidence for most outcomes was low or very low, necessitating cautious interpretation. Finally, some outcomes were supported by only a few studies, resulting in meta-analyses with limited sample sizes and potentially unstable effect estimates. In summary, further well-designed, large-scale studies are required to validate these findings.

At the clinical level, VR-ERI can supplement traditional early rehabilitation, but widespread adoption faces systematic challenges. First, cost remains a key barrier. Although commercial VR prices are falling, expenses may still burden some institutions or patients, with VR for early pain management costing approximately €47.48 (approximately US $51.30) per patient [78]. Policymakers should thus consider including VR in insurance reimbursement or supporting it via dedicated funding. Second, patient vulnerability limits generalizability [79]. Those who are older adults, or have vestibular dysfunction, severe cognitive impairment, or complex life-support needs may be unable to tolerate VR. Health care institutions should form multidisciplinary teams to create standardized screening protocols—focusing on vestibular and cognitive function—and implement a safety system covering pre-, intra-, and postsession phases. Implementation should be phased and individualized—starting at low intensity and adjusted gradually under therapist supervision, while training ward staff and patients in device use. Finally, before broader promotion, safety and feasibility must be validated in heterogeneous critically ill populations [14].

At the policy level, relevant departments should establish a comprehensive support framework by developing evidence-based clinical guidelines for VR rehabilitation that specify indications, contraindications, and protocols, and implementing unified device certification and quality control systems to ensure safe and standardized application. Future research should adopt a phased strategy—priorities include conducting large-scale, rigorous pragmatic trials using blinded outcome assessment, diverse control groups, objective monitoring, and long-term follow-up to minimize bias; creating personalized interventions for specific patient groups, defining a core outcome set, and using objective, standardized assessment tools; and enhancing implementation science to identify optimal rollout pathways across different resource settings—beginning with pilot projects at regional medical centers before scaling to primary care. Concurrently, interdisciplinary collaboration among engineering, rehabilitation medicine, and critical care nursing should be strengthened to jointly advance technology innovation and support the development of a more integrated early rehabilitation system for critically ill patients.

### Conclusions

This review is the first to examine VR rehabilitation effectiveness specifically during early intervention in the ICU. Unlike previous reviews, we focused strictly on the in-ICU early rehabilitation period to establish an evidence base for initiating such care and to minimize the clinical heterogeneity from combining interventions across different care settings. Evidence was synthesized across physiological, psychological, and cognitive domains. Results indicate that VR-ERI shows considerable potential for improving anxiety and subjective sleep quality during ICU treatment, as well as balance, limb motor function, and cognitive function in short-term follow-up. However, effects on pain and objective sleep quality in the ICU remain unclear. Given the high risk of bias in included studies, substantial heterogeneity, and imprecise pooled effect estimates, the evidence certainty for most outcomes is low, and conclusions should be interpreted cautiously. Additionally, establishing a comprehensive safety monitoring system is essential for future clinical translation. This review offers a new perspective on early ICU rehabilitation, suggesting VR technology could serve as a nonpharmacological adjunct in this phase. However, the current evidence is insufficient to support widespread implementation. Clinical translation requires careful consideration of cost and patient suitability, and long-term benefits need validation through large-scale and rigorous research.

## Supplementary material

10.2196/81865 Multimedia Appendix 1 Detailed search strategy.

10.2196/81865 Multimedia Appendix 2 Excluded literature and reasons.

10.2196/81865 Multimedia Appendix 3 Characteristics of included studies.

10.2196/81865 Multimedia Appendix 4 Dual Revised Cochrane Risk-of-Bias tool for randomized trials assessment process with interrater consistency.

10.2196/81865 Multimedia Appendix 5 Grading of Recommendations Assessment, Development, and Evaluation assessment results.

10.2196/81865 Checklist 1 PRISMA checklist for abstracts.

10.2196/81865 Checklist 2 PRISMA-S checklist.

10.2196/81865 Checklist 3 PRISMA checklist.

## Abbreviations

CAM: Confusion Assessment Method
CNKI: China National Knowledge Internet
GRADE: Grading of Recommendations Assessment, Development, and Evaluation
ICU: intensive care unit
MoCA: Montreal Cognitive Assessment
PI: prediction interval
PICOS: Population, Intervention, Comparison, Outcome, and Study Design
PICS: post-intensive care syndrome
PRISMA: Preferred Reporting Items for Systematic Reviews and Meta-Analysis
PRISMA-S: Preferred Reporting Items for Systematic Reviews and Meta-Analyses literature search extension
RCSQ: Richards-Campbell Sleep Questionnaire
RCT: randomized controlled trials
RoB 2: revised Cochrane Risk of Bias tool
SinoMed: SinoMed Service System
SMD: standardized mean difference
TST: total sleep time
VIP: VIP Database
VR: virtual reality
VR-ERI: virtual reality–based early rehabilitation intervention
WASO: wake after sleep onset
