# Supplementary material for: Effectiveness of Virtual Reality–Based Early Rehabilitation Strategies on Pain, Sleep, Anxiety, Balance, Cognition, and Limb Motor Function in Adult Intensive Care Unit Patients: Systematic Review and Meta-Analysis of Randomized Controlled Trials
Source: J Med Internet Res. 2026 Mar 6;28:e81865. doi: 10.2196/81865 (PMC12978899; doi:10.2196/81865)
Supplement: Multimedia Appendix 1 [file jmir-v28-e81865-s001.docx]

Search Strategies (Total Records Identified: N=3,737)

The literature search was conducted and reported in detail following the PRISMA-S Checklist, as required by the PRISMA 2020 guidelines. The following databases were searched: PubMed (n=81), Ovid Embase (n=668), Web of Science (n=1,583), Cochrane Library (n=645), ProQuest (n=347), Ovid Medline (n=262), CNKI (n=28), WanFang Data (n=36), VIP (n=49), and Sinomed (n=38).

| **Database** | **Query** | **Hits** |
| --- | --- | --- |
| **PubMed**  (https://www.ncbi.nlm.nih.gov/)  Date of search (2025.10.05) | #1 **Search: "Virtual Reality"[Mesh] Sort by: Most Recent**  #2 Search:  'virtual reality'[Title/Abstract] OR 'VR'[Title/Abstract] OR 'virtual reality exposure'[Title/Abstract] OR 'virtual reality distraction'[Title/Abstract] OR 'virtual reality immersive'[Title/Abstract] OR 'virtual environment'[Title/Abstract] OR 'virtual game'[Title/Abstract] OR 'instructional virtual reality'[Title/Abstract]  #3 #1 OR #2  #4 **Search: "Intensive Care Units"[Mesh] Sort by: Most Recent**  #5 Search: 'Intensive Care Unit'[Title/Abstract] OR 'ICU'[Title/Abstract] OR 'intensive care'[Title/Abstract] OR 'critical care*'[Title/Abstract] OR 'critical ill*'[Title/Abstract] OR 'critical illness'[Title/Abstract] OR 'intensive care units'[Title/Abstract] OR 'critical care nursing'[Title/Abstract] OR 'severely ill patient'[Title/Abstract]  #6 #4 OR #5  #7 **Search: "Delirium"[Mesh] Sort by: Most Recent**  #8 Search: 'delirium'[Title/Abstract] OR 'subacute delirium'[Title/Abstract] OR 'mixed origin delirium'[Title/Abstract] OR 'Emergence Delirium'[Title/Abstract] OR 'Agitated Emergence'[Title/Abstract] OR 'Emergence Excitement'[Title/Abstract] OR 'Postanesthetic Excitement'[Title/Abstract] OR 'Anesthesia Emergence Delirium'[Title/Abstract] OR 'Postoperative Delirium'[Title/Abstract] OR 'Post Operative Delirium'[Title/Abstract] OR 'Disorientation'[Title/Abstract] OR 'Bewilderment'[Title/Abstract] OR 'Mental Disorder'[Title/Abstract] OR 'Psychosis'[Title/Abstract]  #9 **Search: "Cognition"[Mesh] Sort by: Most Recent**  #10 Search: 'Cognitions'[Title/Abstract] OR ' Cognitive Function'[Title/Abstract]  #11 **Search: "Pain"[Mesh] Sort by: Most Recent**  #12 Search: 'Suffering*, Physical'[Title/Abstract] OR 'Ache*'[Title/Abstract]  #13 **Search: "Anxiety"[Mesh] Sort by: Most Recent**  #14 Search: 'Angst'[Title/Abstract] OR 'Nervousness'[Title/Abstract] OR 'Hypervigilance'[Title/Abstract] OR 'Social Anxiety*'[Title/Abstract] OR 'Anxiousness'[Title/Abstract]  #15 **Search: "****Depression"[Mesh] Sort by: Most Recent**  #16 Search: 'Depressive Symptom*'[Title/Abstract] OR 'Emotional Depression'[Title/Abstract]  #17 **Search: "****Stress Disorders, Post-Traumatic"[Mesh] Sort by: Most Recent**  #18 Search: 'Post-Traumatic Stress Disorder'[Title/Abstract] OR 'Neuroses, Post-Traumat'[Title/Abstract] OR 'PTSD'[Title/Abstract] OR 'posttraumatic stress disorder*'[Title/Abstract] OR 'Moral Injury*'[Title/Abstract] OR 'Posttraumatic Neuroses'[Title/Abstract]  #19 **Search: "Sleep"[Mesh] Sort by: Most Recent**  #20 Search: 'Sleep'[Title/Abstract] OR 'Sleep Quality'[Title/Abstract] OR 'Sleep Pattern'[Title/Abstract] OR 'Sleep Behavior'[Title/Abstract] OR 'Sleep Duration'[Title/Abstract] OR 'Sleep Efficiency'[Title/Abstract] OR 'Sleep Latency'[Title/Abstract] OR 'Sleep Wake Cycle'[Title/Abstract] OR 'Sleep Architecture'[Title/Abstract] OR 'REM Sleep'[Title/Abstract] OR 'Non-REM Sleep'[Title/Abstract] OR 'Slow Wave Sleep'[Title/Abstract] OR 'Sleep Disorders'[Title/Abstract] OR 'Insomnia'[Title/Abstract] OR 'Hypersomnia'[Title/Abstract] OR 'Parasomnia'[Title/Abstract] OR 'Sleep Apnea'[Title/Abstract] OR 'Sleep Related Breathing Disorder'[Title/Abstract] OR 'Circadian Rhythm Sleep Disorder'[Title/Abstract] OR 'Restless Legs Syndrome'[Title/Abstract] OR 'Narcolepsy'[Title/Abstract] OR 'Sleep Walking'[Title/Abstract] OR 'Night Terror'[Title/Abstract] OR 'Sleep Talking'[Title/Abstract] OR 'Sleep Disturbance'[Title/Abstract] OR 'Sleep Deprivation'[Title/Abstract] Sort by: Most Recent  #21 **Search: "Postural Balance"[Mesh] Sort by: Most Recent**  #22 Search: "Postural Balance"[Title/Abstract] OR "Postural Control"[Title/Abstract] OR "Postural Stability"[Title/Abstract] OR "Body Balance"[Title/Abstract] OR "Static Balance"[Title/Abstract] OR "Upright Balance"[Title/Abstract] OR "Standing Balance"[Title/Abstract] OR "Postural Equilibrium"[Title/Abstract] OR "Postural Sway"[Title/Abstract] OR "Balance Control"[Title/Abstract] OR "Balance Ability"[Title/Abstract] OR "Posturography"[Title/Abstract] OR "Postural Reaction"[Title/Abstract] OR "Postural Adjustment"[Title/Abstract] OR "Postural Tone"[Title/Abstract] OR "Postural Orientation"[Title/Abstract] OR "Postural Alignment"[Title/Abstract] OR "Postural Coordination"[Title/Abstract] OR "Postural Instability"[Title/Abstract] OR "Balance Disorder"[Title/Abstract] OR "Balance Impairment"[Title/Abstract] OR "Balance Dysfunction"[Title/Abstract] OR "Loss of Balance"[Title/Abstract] OR "Balance Deficit"[Title/Abstract] Sort by: Most Recent  #23 **Search: "Quality of Life"[Mesh] Sort by: Most Recent**  #24 Search: "Quality of Life"[Title/Abstract] OR "QoL"[Title/Abstract] OR "Health-Related Quality of Life"[Title/Abstract] OR "HRQoL"[Title/Abstract] OR "Well-Being"[Title/Abstract] OR "Life Satisfaction"[Title/Abstract] OR "Activities of Daily Living"[Title/Abstract] OR "ADL"[Title/Abstract] OR "Patient-Reported Outcomes"[Title/Abstract] OR "PRO*"[Title/Abstract] OR "Health Status"[Title/Abstract] OR "Social Functioning"[Title/Abstract] OR "Economic Well-Being"[Title/Abstract] Sort by: Most Recent  #25 **Search: #7 OR #8 OR #9 OR #10 OR #11 OR #12 OR #13 OR #14 OR #15 OR #16 OR #17 OR #18 OR # 19 OR #20 OR #21 OR #22 OR #23 OR #24**  #26 **Search: #3 AND #6 AND #25** | 9,322  34,145  35,450  119,141  304,683  333,305  14,638  94,609  221,425  73,214  494,018  29,655  130,148  13,751  174,106  85,172  46,560  54,642  109,449  276,108  31,457  28,632  312,219  1,015,520  2,416,470  81 |
| **Ovid Embase**  (https://www.embase.com)  Date of search (2025.10.05) | #1  **'virtual reality'/exp**  #2 'virtual reality':ab,ti OR 'vr':ab,ti OR 'virtual reality exposure':ab,ti OR 'virtual reality distraction':ab,ti OR 'virtual reality immersive':ab,ti OR 'virtual environment':ab,ti OR 'virtual game':ab,ti OR 'instructional virtual reality':ab,ti  #3 **#1 OR #2**  #4 **'intensive care unit'/exp**  #5 'intensive care unit':ab,ti OR 'icu':ab,ti OR 'intensive care':ab,ti OR 'critical care*':ab,ti OR 'critical ill*':ab,ti OR 'critical illness':ab,ti OR 'intensive care units':ab,ti OR 'critical care nursing':ab,ti OR 'severely ill patient':ab,ti  #6 **#4 OR #5**  #7 **'delirium'/exp**  #8 'delirium':ab,ti OR 'subacute delirium':ab,ti OR 'mixed origin delirium':ab,ti OR 'emergence delirium':ab,ti OR 'agitated emergence':ab,ti OR 'emergence excitement':ab,ti OR 'postanesthetic excitement':ab,ti OR 'anesthesia emergence delirium':ab,ti OR 'postoperative delirium':ab,ti OR 'post operative delirium':ab,ti OR 'disorientation':ab,ti OR 'bewilderment':ab,ti OR 'mental disorder':ab,ti OR 'psychosis':ab,ti  #9 **'cognition'/exp**  #10 'cognitions':ab,ti OR 'cognitive function':ab,ti  #11 **'pain'/exp**  #12 'suffering*, physical':ab,ti OR 'ache*':ab,ti  #13 **'anxiety'/exp**  #14 'angst':ab,ti OR 'nervousness':ab,ti OR 'hypervigilance':ab,ti OR 'social anxiety*':ab,ti OR 'anxiousness':ab,ti  #15 **'depression'/exp**  #16 'depressive symptom*':ab,ti OR 'emotional depression':ab,ti  #17 **'post-traumatic stress disorder'/exp**  #18 'post-traumatic stress disorder':ab,ti OR 'neuroses, post-traumat':ab,ti OR 'ptsd':ab,ti OR 'posttraumatic stress disorder*':ab,ti OR 'moral injury*':ab,ti OR 'posttraumatic neuroses':ab,ti  #19 **'sleep'/exp**  #20 'sleep':ab,ti OR 'sleep quality':ab,ti OR 'sleep pattern':ab,ti OR 'sleep behavior':ab,ti OR 'sleep duration':ab,ti OR 'sleep efficiency':ab,ti OR 'sleep latency':ab,ti OR 'sleep wake cycle':ab,ti OR 'sleep architecture':ab,ti OR 'rem sleep':ab,ti OR 'non-rem sleep':ab,ti OR 'slow wave sleep':ab,ti OR 'sleep disorders':ab,ti OR 'insomnia':ab,ti OR 'hypersomnia':ab,ti OR 'parasomnia':ab,ti OR 'sleep apnea':ab,ti OR 'sleep related breathing disorder':ab,ti OR 'circadian rhythm sleep disorder':ab,ti OR 'restless legs syndrome':ab,ti OR 'narcolepsy':ab,ti OR 'sleep walking':ab,ti OR 'night terror':ab,ti OR 'sleep talking':ab,ti OR 'sleep disturbance':ab,ti OR 'sleep deprivation':ab,ti  #21 **'postural balance'/exp**  #22 'postural balance':ab,ti OR 'postural control':ab,ti OR 'postural stability':ab,ti OR 'body balance':ab,ti OR 'static balance':ab,ti OR 'upright balance':ab,ti OR 'standing balance':ab,ti OR 'postural equilibrium':ab,ti OR 'postural sway':ab,ti OR 'balance control':ab,ti OR 'balance ability':ab,ti OR 'posturography':ab,ti OR 'postural reaction':ab,ti OR 'postural adjustment':ab,ti OR 'postural tone':ab,ti OR 'postural orientation':ab,ti OR 'postural alignment':ab,ti OR 'postural coordination':ab,ti OR 'postural instability':ab,ti OR 'balance disorder':ab,ti OR 'balance impairment':ab,ti OR 'balance dysfunction':ab,ti OR 'loss of balance':ab,ti OR 'balance deficit':ab,ti  #23 **'quality of life'/exp**  #24 'quality of life':ab,ti OR 'qol':ab,ti OR 'health-related quality of life':ab,ti OR 'hrqol':ab,ti OR 'well-being':ab,ti OR 'life satisfaction':ab,ti OR 'activities of daily living':ab,ti OR 'adl':ab,ti OR 'patient-reported outcomes':ab,ti OR 'pro*':ab,ti OR 'health status':ab,ti OR 'social functioning':ab,ti OR 'economic well-being':ab,ti  #25 **#7 OR #8 OR #9 OR #10 OR #11 OR #12 OR #13 OR #14 OR #15 OR #16 OR #17 OR #18 OR #19 OR #20 OR #21 OR #22 OR #23 OR #24**  #26 **#3 AND #6 AND #25**  ***["/exp" indicates expanding to subordinate subject terms (explode), while "ab,ti" is used to limit the search terms to the abstract (ab) and title (ti) fields for retrieval.]*** | 37,894  44,540  57,634  386,063  493,310  618,584  53,140  139,265  3,656,228  101,399  2,018,604  38,703  384,808  18,300  786,026  111,924  99,820  69,074  354,786  431,364  27,933  38,041  840,084  28,511,184  30,799,773  668 |
| **Web of Science**  (https://www.webofscience.com/wos/woscc/basic-search)  Date of search (2025.10.05) | 1 TS=('virtual reality' OR 'VR' OR 'virtual reality exposure' OR 'virtual reality distraction' OR 'virtual reality immersive' OR 'virtual environment' OR 'virtual game' OR 'instructional virtual reality')  2 TS=('Intensive Care Unit' OR 'ICU' OR 'intensive care' OR 'critical care*' OR 'critical ill*' OR 'critical illness' OR 'intensive care units' OR 'critical care nursing' OR 'severely ill patient')  3 TS=('delirium' OR 'subacute delirium' OR 'mixed origin delirium' OR 'Emergence Delirium' OR 'Agitated Emergence' OR 'Emergence Excitement' OR 'Postanesthetic Excitement' OR 'Anesthesia Emergence Delirium' OR 'Postoperative Delirium' OR 'Post Operative Delirium' OR 'Disorientation' OR 'Bewilderment' OR 'Mental Disorder' OR 'Psychosis')  4 TS=('Cognitions' OR 'Cognitive Function')  5 TS=('Suffering*, Physical' OR 'Ache*')  6 TS=('Angst' OR 'Nervousness' OR 'Hypervigilance' OR 'Social Anxiety*' OR 'Anxiousness')  7 TS=('Depressive Symptom*' OR 'Emotional Depression')  8 TS=('Post-Traumatic Stress Disorder' OR 'Neuroses, Post-Traumat' OR 'PTSD' OR 'posttraumatic stress disorder*' OR 'Moral Injury*' OR 'Posttraumatic Neuroses')  9 TS=('Sleep' OR 'Sleep Quality' OR 'Sleep Pattern' OR 'Sleep Behavior' OR 'Sleep Duration' OR 'Sleep Efficiency' OR 'Sleep Latency' OR 'Sleep Wake Cycle' OR 'Sleep Architecture' OR 'REM Sleep' OR 'Non-REM Sleep' OR 'Slow Wave Sleep' OR 'Sleep Disorders' OR 'Insomnia' OR 'Hypersomnia' OR 'Parasomnia' OR 'Sleep Apnea' OR 'Sleep Related Breathing Disorder' OR 'Circadian Rhythm Sleep Disorder' OR 'Restless Legs Syndrome' OR 'Narcolepsy' OR 'Sleep Walking' OR 'Night Terror' OR 'Sleep Talking' OR 'Sleep Disturbance' OR 'Sleep Deprivation')  10 TS=('Postural Balance' OR 'Postural Control' OR 'Postural Stability' OR 'Body Balance' OR 'Static Balance' OR 'Upright Balance' OR 'Standing Balance' OR 'Postural Equilibrium' OR 'Postural Sway' OR 'Balance Control' OR 'Balance Ability' OR 'Posturography' OR 'Postural Reaction' OR 'Postural Adjustment' OR 'Postural Tone' OR 'Postural Orientation' OR 'Postural Alignment' OR 'Postural Coordination' OR 'Postural Instability' OR 'Balance Disorder' OR 'Balance Impairment' OR 'Balance Dysfunction' OR 'Loss of Balance' OR 'Balance Deficit')  11 TS=('Quality of Life' OR 'QoL' OR 'Health-Related Quality of Life' OR 'HRQoL' OR 'Well-Being' OR 'Life Satisfaction' OR 'Activities of Daily Living' OR 'ADL' OR 'Patient-Reported Outcomes' OR 'PRO*' OR 'Health Status' OR 'Social Functioning' OR 'Economic Well-Being')  12 #11 OR #10 OR #9 OR #8 OR #7 OR #6 OR #5 OR #4 OR #3  13 #12 AND #1 AND #2  ***[The TS search qualifier indicates searching within the fields of Title, Abstract, and Author Keywords.]*** | 197,701  592,994  352,952  458,348  52,136  105,617  200,703  94,436  388,560  479,959  46,100,420  46,557,677  1,583 |
| **Cochrane Library**  (http://www-cochranelibrary-com-443.ca.ilibs.cn/)  Date of search (2025.10.05) | #1 ('virtual reality' OR 'VR' OR 'virtual reality exposure' OR 'virtual reality distraction' OR 'virtual reality immersive' OR 'virtual environment' OR 'virtual game' OR 'instructional virtual reality' 'Virtual Reality Immersion Therapy*' OR 'Virtual Reality Therapy*''virtual reality' OR 'VR' OR 'virtual reality exposure' OR 'virtual reality distraction' OR 'virtual reality immersive' OR 'virtual environment' OR 'virtual game' OR 'instructional virtual reality' 'Virtual Reality Immersion Therapy*' OR 'Virtual Reality Therapy*'):ti,ab,kw  #2 ('Intensive Care Unit' OR 'ICU' OR 'intensive care' OR 'critical care*' OR 'critical ill*' OR 'intensive care units' OR 'critical care nursing' OR 'severely ill patient'):ti,ab,kw  #3 ('delirium' OR 'subacute delirium' OR 'mixed origin delirium' OR 'Emergence Delirium' OR 'Agitated* Emergence' OR 'Emergence Excitement' OR 'Postanesthetic Excitement' OR 'Postoperative Delirium'):ti,ab,kw  #4 ('Cognition' OR 'Cognitions' OR ' Cognitive Function'):ti,ab,kw  #5 ('Pain' OR 'Suffering*, Physical' OR 'Ache*'):ti,ab,kw  #6 ('Anxiety' OR 'Angst' OR 'Nervousness' OR 'Hypervigilance' OR 'Social Anxiety*' OR 'Anxiousness'):ti,ab,kw  #7 ('Depression' OR 'Depressive Symptom*' OR 'Emotional Depression'):ti,ab,kw  #8 ('Post-Traumatic Stress Disorder' OR 'Neuroses, Post-Traumat' OR 'PTSD' OR 'Posttraumatic Stress Disorder*' OR 'Moral Injury*' OR 'Posttraumatic Neuroses'):ti,ab,kw  #9 ('Sleep' OR 'Sleep Quality' OR 'Sleep Pattern' OR 'Sleep Behavior' OR 'Sleep Duration' OR 'Sleep Efficiency' OR 'Sleep Latency' OR 'Sleep Wake Cycle' OR 'Sleep Architecture' OR 'REM Sleep' OR 'Non-REM Sleep' OR 'Slow Wave Sleep' OR 'Sleep Disorders' OR 'Insomnia' OR 'Hypersomnia' OR 'Parasomnia' OR 'Sleep Apnea' OR 'Sleep Related Breathing Disorder' OR 'Circadian Rhythm Sleep Disorder' OR 'Restless Legs Syndrome' OR 'Narcolepsy' OR 'Sleep Walking' OR 'Night Terror' OR 'Sleep Talking' OR 'Sleep Disturbance' OR 'Sleep Deprivation'):ti,ab,kw  #10 ('Postural Balance' OR 'Postural Control' OR 'Postural Stability' OR 'Body Balance' OR 'Static Balance' OR 'Upright Balance' OR 'Standing Balance' OR 'Postural Equilibrium' OR 'Postural Sway' OR 'Balance Control' OR 'Balance Ability' OR 'Posturography' OR 'Postural Reaction' OR 'Postural Adjustment' OR 'Postural Tone' OR 'Postural Orientation' OR 'Postural Alignment' OR 'Postural Coordination' OR 'Postural Instability' OR 'Balance Disorder' OR 'Balance Impairment' OR 'Balance Dysfunction' OR 'Loss of Balance' OR 'Balance Deficit'):ti,ab,kw  #11 ('Quality of Life' OR 'QoL' OR 'Health-Related Quality of Life' OR 'HRQoL' OR 'Well-Being' OR 'Life Satisfaction' OR 'Activities of Daily Living' OR 'ADL' OR 'Patient-Reported Outcomes' OR 'PRO*' OR 'Health Status' OR 'Social Functioning' OR 'Economic Well-Being'):ti,ab,kw  #12 #11 OR #10 OR #9 OR #8 OR #7 OR #6 OR #5 OR #4 OR #3  #13 #12 AND #1 AND #2  ***[ti,ab,kw represent searching within the title, abstract, and keyword fields.]*** | 11,426  109,707  7,766  121,995  281,466  123,255  128,363  9,761  70,732  62,726  1,608,244  1,745,210  645 |
| **ProQuest**  (https://www.proquest.com/advanced?accountid=13585)  Date of search (2025.10.05) | S1 abstract('virtual reality' OR 'VR' OR 'virtual reality exposure' OR 'virtual reality distraction' OR 'virtual reality immersive' OR 'virtual environment' OR 'virtual game' OR 'instructional virtual reality' 'Virtual Reality Immersion Therapy*' OR 'Virtual Reality Therapy*')  S2 abstract('Intensive Care Unit' OR 'ICU' OR 'intensive care' OR 'critical care*' OR 'critical ill*' OR 'intensive care units' OR 'critical care nursing' OR 'severely ill patient')  S3 abstract('delirium' OR 'subacute delirium' OR 'mixed origin delirium' OR 'Emergence Delirium' OR 'Agitated* Emergence' OR 'Emergence Excitement' OR 'Postanesthetic Excitement' OR 'Postoperative Delirium')  S4 abstract('Cognition' OR 'Cognitions' OR ' Cognitive Function')  S5 abstract('Pain' OR 'Suffering*, Physical' OR 'Ache*')  S6 abstract('Anxiety' OR 'Angst' OR 'Nervousness' OR 'Hypervigilance' OR 'Social Anxiety*' OR 'Anxiousness' )  S7 abstract('Depression' OR 'Depressive Symptom*' OR 'Emotional Depression' )  S8 abstract('Post-Traumatic Stress Disorder' OR 'Neuroses, Post-Traumat' OR 'PTSD' OR 'Posttraumatic Stress Disorder*' OR 'Moral Injury*' OR 'Posttraumatic Neuroses')  S9 abstract('Sleep' OR 'Sleep Quality' OR 'Sleep Pattern' OR 'Sleep Behavior' OR 'Sleep Duration' OR 'Sleep Efficiency' OR 'Sleep Latency' OR 'Sleep Wake Cycle' OR 'Sleep Architecture' OR 'REM Sleep' OR 'Non-REM Sleep' OR 'Slow Wave Sleep' OR 'Sleep Disorders' OR 'Insomnia' OR 'Hypersomnia' OR 'Parasomnia' OR 'Sleep Apnea' OR 'Sleep Related Breathing Disorder' OR 'Circadian Rhythm Sleep Disorder' OR 'Restless Legs Syndrome' OR 'Narcolepsy' OR 'Sleep Walking' OR 'Night Terror' OR 'Sleep Talking' OR 'Sleep Disturbance' OR 'Sleep Deprivation')  S10 abstract('Postural Balance' OR 'Postural Control' OR 'Postural Stability' OR 'Body Balance' OR 'Static Balance' OR 'Upright Balance' OR 'Standing Balance' OR 'Postural Equilibrium' OR 'Postural Sway' OR 'Balance Control' OR 'Balance Ability' OR 'Posturography' OR 'Postural Reaction' OR 'Postural Adjustment' OR 'Postural Tone' OR 'Postural Orientation' OR 'Postural Alignment' OR 'Postural Coordination' OR 'Postural Instability' OR 'Balance Disorder' OR 'Balance Impairment' OR 'Balance Dysfunction' OR 'Loss of Balance' OR 'Balance Deficit')  S11 abstract('Quality of Life' OR 'QoL' OR 'Health-Related Quality of Life' OR 'HRQoL' OR 'Well-Being' OR 'Life Satisfaction' OR 'Activities of Daily Living' OR 'ADL' OR 'Patient-Reported Outcomes' OR 'PRO*' OR 'Health Status' OR 'Social Functioning' OR 'Economic Well-Being')  S12 [S3] OR [S4] OR [S5] OR [S6] OR [S7] OR [S8] OR [S9] OR [S10] OR [S11]  S13 [S1] AND [S2] AND [S12] | 44,797  106,334  4,299  83,116  154,921  107,432  119,282  12,237  54,099  60,854  8,178,287  8,364,986  347 |
| **Ovid Medline**  (http://www.ncbi.nlm.nih.gov/medline)  Date of search (2025.10.05) | 1 ('virtual reality' or 'VR' or 'virtual reality exposure' or 'virtual reality distraction' or 'virtual reality immersive' or 'virtual environment' or 'virtual game' or 'instructional virtual reality' or 'Virtual Reality Immersion Therapy*' or 'Virtual Reality Therapy*').mp. ***[mp=title, book title, abstract, original title, name of substance word, subject heading word, floating sub-heading word, keyword heading word, organism supplementary concept word, protocol supplementary concept word, rare disease supplementary concept word, unique identifier, synonyms, population supplementary concept word, anatomy supplementary concept word]***  2 ('Intensive Care Unit' or 'ICU' or 'intensive care' or 'critical care*' or 'critical ill*' or 'intensive care units' or 'critical care nursing' or 'severely ill patient').mp. ***[mp=title, book title, abstract, original title, name of substance word, subject heading word, floating sub-heading word, keyword heading word, organism supplementary concept word, protocol supplementary concept word, rare disease supplementary concept word, unique identifier, synonyms, population supplementary concept word, anatomy supplementary concept word]***  3 ('delirium' or 'subacute delirium' or 'mixed origin delirium' or 'Emergence Delirium' or 'Agitated* Emergence' or 'Emergence Excitement' or 'Postanesthetic Excitement' or 'Postoperative Delirium').mp. ***[mp=title, book title, abstract, original title, name of substance word, subject heading word, floating sub-heading word, keyword heading word, organism supplementary concept word, protocol supplementary concept word, rare disease supplementary concept word, unique identifier, synonyms, population supplementary concept word, anatomy supplementary concept word]***  4 ('Cognition' or 'Cognitions' or ' Cognitive Function').mp. ***[mp=title, book title, abstract, original title, name of substance word, subject heading word, floating sub-heading word, keyword heading word, organism supplementary concept word, protocol supplementary concept word, rare disease supplementary concept word, unique identifier, synonyms, population supplementary concept word, anatomy supplementary concept word]***  5 ('Pain' or 'Suffering*, Physical' or 'Ache*').mp. ***[mp=title, book title, abstract, original title, name of substance word, subject heading word, floating sub-heading word, keyword heading word, organism supplementary concept word, protocol supplementary concept word, rare disease supplementary concept word, unique identifier, synonyms, population supplementary concept word, anatomy supplementary concept word]***  6 ('Anxiety' or 'Angst' or 'Nervousness' or 'Hypervigilance' or 'Social Anxiety*' or 'Anxiousness').mp. ***[mp=title, book title, abstract, original title, name of substance word, subject heading word, floating sub-heading word, keyword heading word, organism supplementary concept word, protocol supplementary concept word, rare disease supplementary concept word, unique identifier, synonyms, population supplementary concept word, anatomy supplementary concept word]***  7 ('Depression' or 'Depressive Symptom*' or 'Emotional Depression').mp. ***[mp=title, book title, abstract, original title, name of substance word, subject heading word, floating sub-heading word, keyword heading word, organism supplementary concept word, protocol supplementary concept word, rare disease supplementary concept word, unique identifier, synonyms, population supplementary concept word, anatomy supplementary concept word]***  8 ('Post-Traumatic Stress Disorder' or 'Neuroses, Post-Traumat' or 'PTSD' or 'Posttraumatic Stress Disorder*' or 'Moral Injury*' or 'Posttraumatic Neuroses').mp. ***[mp=title, book title, abstract, original title, name of substance word, subject heading word, floating sub-heading word, keyword heading word, organism supplementary concept word, protocol supplementary concept word, rare disease supplementary concept word, unique identifier, synonyms, population supplementary concept word, anatomy supplementary concept word]***  9 ('Sleep' or 'Sleep Quality' or 'Sleep Pattern' or 'Sleep Behavior' or 'Sleep Duration' or 'Sleep Efficiency' or 'Sleep Latency' or 'Sleep Wake Cycle' or 'Sleep Architecture' or 'REM Sleep' or 'Non-REM Sleep' or 'Slow Wave Sleep' or 'Sleep Disorders' or 'Insomnia' or 'Hypersomnia' or 'Parasomnia' or 'Sleep Apnea' or 'Sleep Related Breathing Disorder' or 'Circadian Rhythm Sleep Disorder' or 'Restless Legs Syndrome' or 'Narcolepsy' or 'Sleep Walking' or 'Night Terror' or 'Sleep Talking' or 'Sleep Disturbance' or 'Sleep Deprivation').mp. ***[mp=title, book title, abstract, original title, name of substance word, subject heading word, floating sub-heading word, keyword heading word, organism supplementary concept word, protocol supplementary concept word, rare disease supplementary concept word, unique identifier, synonyms, population supplementary concept word, anatomy supplementary concept word]***  10 ('Postural Balance' or 'Postural Control' or 'Postural Stability' or 'Body Balance' or 'Static Balance' or 'Upright Balance' or 'Standing Balance' or 'Postural Equilibrium' or 'Postural Sway' or 'Balance Control' or 'Balance Ability' or 'Posturography' or 'Postural Reaction' or 'Postural Adjustment' or 'Postural Tone' or 'Postural Orientation' or 'Postural Alignment' or 'Postural Coordination' or 'Postural Instability' or 'Balance Disorder' or 'Balance Impairment' or 'Balance Dysfunction' or 'Loss of Balance' or 'Balance Deficit').mp. ***[mp=title, book title, abstract, original title, name of substance word, subject heading word, floating sub-heading word, keyword heading word, organism supplementary concept word, protocol supplementary concept word, rare disease supplementary concept word, unique identifier, synonyms, population supplementary concept word, anatomy supplementary concept word]***  11 ('Quality of Life' or 'QoL' or 'Health-Related Quality of Life' or 'HRQoL' or 'Well-Being' or 'Life Satisfaction' or 'Activities of Daily Living' or 'ADL' or 'Patient-Reported Outcomes' or 'PRO*' or 'Health Status' or 'Social Functioning' or 'Economic Well-Being').mp. ***[mp=title, book title, abstract, original title, name of substance word, subject heading word, floating sub-heading word, keyword heading word, organism supplementary concept word, protocol supplementary concept word, rare disease supplementary concept word, unique identifier, synonyms, population supplementary concept word, anatomy supplementary concept word]***  12 3 or 4 or 5 or 6 or 7 or 8 or 9 or 10 or 11  13 1 and 2 and 12 | 35,103  361,625  28,526  321,773  1,020,466  371,830  582,854  54,620  302,410  46,423  24,623,955  25,311,896  262 |
| **CNKI**  (https://www.cnki.net/)  Date of search (2025.10.05) | #1 SU%='虚拟现实' OR SU%='虚拟环境' OR SU%='虚拟游戏' OR SU%='虚拟化' OR SU%='虚拟机' OR SU%='虚拟干预' OR SU%='VR' OR SU%='虚拟技术'  (#1 SU%='virtual reality' OR SU%='virtual environment' OR SU%='virtual game' OR SU%='virtualization' OR SU%='virtual machine' OR SU%='virtual intervention' OR SU%='VR' OR SU%='virtual technology')  #2 SU%='重症监护' OR SU%='重症医学科' OR SU%='ICU' OR SU%='危重患者' OR SU%='危重病人' OR SU%='ICU 患者' OR SU%='ICU 病人' OR SU%='重症患者' OR SU%='重症病人'  (#2 SU%='intensive care' OR SU%='department of critical care medicine' OR SU%='ICU' OR SU%='critically ill patients' OR SU%='critically ill people' OR SU%='ICU patients' OR SU%='ICU patients' OR SU%='severe patients' OR SU%='severe cases')  #3 SU%='谵妄' OR SU%='谵语' OR SU%='意识模糊' OR SU%='精神失常' OR SU%='精神错乱'  (#3 SU%='delirium' OR SU%='delirious talk' OR SU%='confusion of consciousness' OR SU%='mental disorder' OR SU%='mental confusion')  #4 SU%='认知' OR SU%='认知功能' OR SU%='认知障碍' OR SU%='主观认知障碍' OR SU%='轻度认知障碍'  (#4 SU%='cognition' OR SU%='cognitive function' OR SU%='cognitive impairment' OR SU%='subjective cognitive impairment' OR SU%='mild cognitive impairment')  #5 SU%='疼痛' OR SU%='镇痛' OR SU%='术后疼痛'  (#5 SU%='pain' OR SU%='analgesia' OR SU%='postoperative pain')  #6 SU%='焦虑' OR SU%='情志异常' OR SU%='负性情绪' OR SU%='负面情绪'  (#6 SU%='anxiety' OR SU%='emotional disturbance' OR SU%='negative emotion' OR SU%='negative feelings')  #7 SU%='抑郁' OR SU%='情绪抑郁'  (#7 SU%='depression' OR SU%='depressive emotion')  #8 SU%='创伤后应激障碍' OR SU%='创伤后应激' OR SU%='应激障碍' OR SU%='精神障碍'  #9 SU%='睡眠障碍' OR SU%='失眠' OR SU%='早醒' OR SU%='多梦'  (#9 SU%='Sleep Disorders' OR SU%='Insomnia' OR SU%='Early Awakening' OR SU%='Dreaminess' )  #10 SU%='平衡能力' OR SU%='运动能力' OR SU%='肢体平衡' OR SU%='平衡力'  (#10 SU%='Balance Ability' OR SU%='Motor Ability' OR SU%='Limb Balance' OR SU%='Balance Capacity')  #11 SU%='生活质量' OR SU%='生命质量' OR SU%='康复质量'  (#11 SU%='Quality of Life' OR SU%='Quality of Life' OR SU%='Quality of Rehabilitation' )  #12 #3 OR #4 OR #5 OR #6 OR #7 OR #8 OR #9 OR #10 OR #11  #13 #1 AND #2 AND #12  ***["SU" represents the subject field, which is used to retrieve the thematic content of documents.]*** | 147,636  148,197  12,132  696,236  494,139  234,971  194,445  35,789  68,711  33,366  325,721  1,838,079  28 |
| **WanFang**  (https://w.wanfangdata.com.cn/index.html?index=true)  Date of search (2025.10.05) | #1主题:(虚拟现实 OR 虚拟环境 OR 虚拟游戏 OR 虚拟化 OR 虚拟机 OR 虚拟干预 OR VR OR 虚拟技术)  (#1 Theme: (Virtual Reality OR Virtual Environment OR Virtual Game OR Virtualization OR Virtual Machine OR Virtual Intervention OR VR OR Virtual Technology))  #2主题:(重症监护 OR 重症医学科 OR ICU OR 危重患者 OR 危重病人 OR ICU 患者 OR ICU 病人 OR 重症患者 OR 重症病人)  #2 Theme: (Intensive Care OR Department of Critical Care Medicine OR ICU OR Critically Ill Patients OR Critically Ill People OR ICU Patients OR ICU Patients OR Severe Patients OR Severe Cases)  #3主题:(谵妄 OR 谵语 OR 意识模糊 OR 精神失常 OR 精神错乱)  (#3 Theme: (Delirium OR Delirious Talk OR Confusion of Consciousness OR Mental Disorder OR Mental Confusion))  #4主题: (认知OR 认知功能 OR 认知障碍 OR 主观认知障碍 OR 轻度认知障碍)  (#4 Theme: (Cognition OR Cognitive Function OR Cognitive Impairment OR Subjective Cognitive Impairment OR Mild Cognitive Impairment))  #5主题: (疼痛 OR 镇痛 OR 术后疼痛 OR 术后镇痛)  (#5 Theme: (Pain OR Analgesia OR Postoperative Pain OR Postoperative Analgesia))  #6主题:(焦虑 OR 情志异常 OR 负性情绪 OR 负面情绪)  (#6 Theme: (Anxiety OR Emotional Disturbance OR Negative Emotion OR Negative Feelings))  #7主题:(抑郁 OR 情绪抑郁 OR 抑郁症状)  (#7 Theme: (Depression OR Depressive Emotion OR Depressive Symptoms))  #8主题:(创伤后应激障碍 OR 创伤后应激 OR 应激障碍 OR 精神障碍)  (#8 Theme: (Post-Traumatic Stress Disorder OR Post-Traumatic Stress OR Stress Disorder OR Mental Disorder))  #9 主题:(失眠 OR 睡眠障碍 OR 早醒 OR 多梦)  (#9 Theme: (Insomnia OR Sleep Disorders OR Early Morning Awakening OR Excessive Dreaming))  #10 主题:(平衡能力 OR 运动能力 OR 肢体平衡 OR 平衡力)  (#10 Theme: (Balance Ability OR Motor Ability OR Limb Balance OR Balance Capacity)  #11 主题:(生活质量 OR 生命质量 OR 康复质量)  (#11 Theme: (Quality of Life OR Quality of Life OR Quality of Rehabilitation)  #12 #3 OR #4 OR #5 OR #6 OR #7 OR #8 OR #9 OR #10 OR #11  #13 #1 AND #2 AND #12 | 481,331  545,485  67,146  72,244  1,554,574  688,956  804,959  84,916  121,939  841,806  1,412,061  1,678,937  36 |
| **VIP**  (https://qikan.cqvip.com/)  Date of search (2025.10.05) | #1 U=(虚拟现实 OR 虚拟环境 OR 虚拟游戏 OR 虚拟化 OR 虚拟机 OR 虚拟干预 OR VR OR 虚拟技术)  (#1 U = (Virtual Reality OR Virtual Environment OR Virtual Game OR Virtualization OR Virtual Machine OR Virtual Intervention OR VR OR Virtual Technology))  #2 U=(重症监护 OR 重症医学科 OR ICU OR 危重患者 OR 危重病人 OR ICU 患者 OR ICU 病人 OR 重症患者 OR 重症病人)  (#2 U = (Intensive Care OR Department of Critical Care Medicine OR ICU OR Critically Ill Patients OR Critically Ill People OR ICU Patients OR ICU Patients OR Severe Patients OR Severe Cases))  #3 U=(谵妄 OR 谵语 OR 意识模糊 OR 精神失常 OR 精神错乱)  (#3 U = (Delirium OR Delirious Talk OR Confusion of Consciousness OR Mental Disorder OR Mental Confusion))  #4 U=(认知 OR 认知功能 OR 认知障碍 OR 主观认知障碍 OR 轻度认知障碍)  (#4 U = (Cognition OR Cognitive Function OR Cognitive Impairment OR Subjective Cognitive Impairment OR Mild Cognitive Impairment))  #5 U=(疼痛 OR 镇痛 OR 术后疼痛 OR 术后镇痛)  (#5 U = (Pain OR Analgesia OR Postoperative Pain OR Postoperative Analgesia))  #6 U=(焦虑 OR 情志异常 OR 负性情绪 OR 负面情绪)  (#6 U = (Anxiety OR Emotional Disturbance OR Negative Emotion OR Negative Feelings))  #7 U=(抑郁 OR 情绪抑郁 OR 抑郁症状)  (#7 U = (Depression OR Depressive Emotion OR Depressive Symptoms))  #8 U=(创伤后应激障碍 OR 创伤后应激 OR 应激障碍 OR 精神障碍)  (#8 U = (Post-Traumatic Stress Disorder OR Post-Traumatic Stress OR Stress Disorder OR Mental Disorder))  #9 U=(失眠 OR 睡眠障碍 OR 早醒 OR 多梦)  (#9 U = (Insomnia OR Sleep Disorders OR Early Morning Awakening OR Excessive Dreaming))  #10 U=(平衡能力 OR 运动能力 OR 肢体平衡 OR 平衡力)  (#10 U = (Balance Ability OR Motor Ability OR Limb Balance OR Balance Capacity))  #11 U=(生活质量 OR 生命质量 OR 康复质量)  (#11 U = (Quality of Life OR Quality of Life OR Quality of Rehabilitation))  #12 #3 OR #4 OR #5 OR #6 OR #7 OR #8 OR #9 OR #10 OR #11  #13 #1 AND #2 AND #12  ***["U" represents any field, used for searching without specifying a particular field, but instead searching across all retrievable fields of the document for records containing the specified keywords.]*** | 193,124  327,195  17,836  771,864  648,351  291,594  237,952  49,699  162,512  122,248  788,031  1,122,896  49 |
| **Sinomed**  ((https://www.sinomed.ac.cn)  Date of search (2025.10.05) | #1 ("(虚拟现实"[常用字段:智能] OR "虚拟环境"[常用字段:智能] OR "虚拟游戏"[常用字段:智能] OR "虚拟化"[常用字段:智能] OR "虚拟机"[常用字段:智能] OR "虚拟干预"[常用字段:智能] OR "VR"[常用字段:智能] OR "虚拟技术)"[常用字段:智能])  (#1 ("Virtual Reality" [Common Field: Intelligence] OR "Virtual Environment" [Common Field: Intelligence] OR "Virtual Game" [Common Field: Intelligence] OR "Virtualization" [Common Field: Intelligence] OR "Virtual Machine" [Common Field: Intelligence] OR "Virtual Intervention" [Common Field: Intelligence] OR "VR" [Common Field: Intelligence] OR "Virtual Technology" [Common Field: Intelligence]))  #2 ("(重症监护"[常用字段:智能] OR "重症医学科"[常用字段:智能] OR "ICU"[常用字段:智能] OR "危重患者"[常用字段:智能] OR "危重病人"[常用字段:智能] OR "ICU 患者"[常用字段:智能] OR "ICU 病人"[常用字段:智能] OR "重症患者"[常用字段:智能] OR "重症病人)"[常用字段:智能])  (#2 ("Intensive Care" [Common Field: Intelligence] OR "Department of Critical Care Medicine" [Common Field: Intelligence] OR "ICU" [Common Field: Intelligence] OR "Critically Ill Patients" [Common Field: Intelligence] OR "Critically Ill People" [Common Field: Intelligence] OR "ICU Patients" [Common Field: Intelligence] OR "ICU Patients" [Common Field: Intelligence] OR "Severe Patients" [Common Field: Intelligence] OR "Severe Cases" [Common Field: Intelligence]))  #3 ("谵妄"[常用字段:智能] OR "谵语"[常用字段:智能] OR "意识模糊"[常用字段:智能] OR "精神失常"[常用字段:智能] OR "精神错乱"[常用字段:智能])  (#3 ("Delirium" [Common Field: Intelligence] OR "Delirious Talk" [Common Field: Intelligence] OR "Confusion of Consciousness" [Common Field: Intelligence] OR "Mental Disorder" [Common Field: Intelligence] OR "Mental Confusion" [Common Field: Intelligence]))  #4 ( "认知"[常用字段:智能] OR "认知功能"[常用字段:智能] OR "认知障碍"[常用字段:智能] OR "主观认知障碍"[常用字段:智能] OR "轻度认知障碍"[常用字段:智能])  (#4 ("Cognition" [Common Field: Intelligence] OR "Cognitive Function" [Common Field: Intelligence] OR "Cognitive Impairment" [Common Field: Intelligence] OR "Subjective Cognitive Impairment" [Common Field: Intelligence] OR "Mild Cognitive Impairment" [Common Field: Intelligence]))  #5 ( "疼痛"[常用字段:智能] OR "镇痛"[常用字段:智能] OR "术后疼痛"[常用字段:智能] OR "术后镇痛"[常用字段:智能])  (#5 ("Pain" [Common Field: Intelligence] OR "Analgesia" [Common Field: Intelligence] OR "Postoperative Pain" [Common Field: Intelligence] OR "Postoperative Analgesia" [Common Field: Intelligence]))  #6 ( "焦虑"[常用字段:智能] OR "情志异常"[常用字段:智能] OR "负性情绪"[常用字段:智能] OR "负面情绪"[常用字段:智能])  #6 ("Anxiety" [Common Field: Intelligence] OR "Emotional Disturbance" [Common Field: Intelligence] OR "Negative Emotion" [Common Field: Intelligence] OR "Negative Feelings" [Common Field: Intelligence])  #7 ( "抑郁"[常用字段:智能] OR "情绪抑郁"[常用字段:智能] OR "抑郁症状"[常用字段:智能])  (#7 ("Depression" [Common Field: Intelligence] OR "Depressive Emotion" [Common Field: Intelligence] OR "Depressive Symptoms" [Common Field: Intelligence]))  #8 ( "创伤后应激障碍"[常用字段:智能] OR "创伤后应激"[常用字段:智能] OR "应激障碍"[常用字段:智能] OR "精神障碍"[常用字段:智能])  (#8 ("Post-Traumatic Stress Disorder" [Common Field: Intelligence] OR "Post-Traumatic Stress" [Common Field: Intelligence] OR "Stress Disorder" [Common Field: Intelligence] OR "Mental Disorder" [Common Field: Intelligence]))  #9 ( "失眠"[常用字段:智能] OR "睡眠障碍"[常用字段:智能] OR "早醒"[常用字段:智能] OR "多梦"[常用字段:智能])  (#9 "Insomnia" [Common Field: Intelligent] OR "Sleep Disorders" [Common Field: Intelligent] OR "Early Morning Awakening" [Common Field: Intelligent] OR "Excessive Dreaming" [Common Field: Intelligent])  #10 ( "平衡能力"[常用字段:智能] OR "运动能力"[常用字段:智能] OR "肢体平衡"[常用字段:智能] OR "平衡力"[常用字段:智能])  (#10 "Balance Ability" [Common Field: Intelligent] OR "Motor Ability" [Common Field: Intelligent] OR "Limb Balance" [Common Field: Intelligent] OR "Balance Capacity" [Common Field: Intelligent])  #11 ( "生活质量"[常用字段:智能] OR "生命质量"[常用字段:智能] OR "康复质量"[常用字段:智能])  (#11 "Quality of Life" [Common Field: Intelligent] OR "Quality of Life" [Common Field: Intelligent] OR "Quality of Rehabilitation" [Common Field: Intelligent])  #12 (#3) OR (#4) OR (#5) OR (#6) OR (#7) OR (#8) OR (#9) OR (#10) OR (#11)  #13 (#1) AND (#2) AND (#12)  ***[Common Field: It is the same as the commonly used fields in each individual database. Intelligent Retrieval: It enables expanded retrieval of search terms and their synonyms (including subject terms).]*** | 40,569  462,688  43,381  682,263  1,703,025  558,564  756,400  1,184,261  227,069  14,742  913,000  2,343,876  38 |
