# Supplementary material for: Effectiveness of Virtual Reality–Based Early Rehabilitation Strategies on Pain, Sleep, Anxiety, Balance, Cognition, and Limb Motor Function in Adult Intensive Care Unit Patients: Systematic Review and Meta-Analysis of Randomized Controlled Trials
Source: J Med Internet Res. 2026 Mar 6;28:e81865. doi: 10.2196/81865 (PMC12978899; doi:10.2196/81865)
Supplement: Multimedia Appendix 2 [file jmir-v28-e81865-s002.docx]

**Excluded studies**

**Table 1: Excluded Studies and Reasons After Full-Text Review**

| **Study: author, year** | **Excluded reason** |
| --- | --- |
| 1. Gerber *et al.,*2017^1^ | feasibility analysis |
| 2. Turon *et al.,*2019^2^ | feasibility analysis |
| 3. Suvajdzic *et al.,*2018^3^ | feasibility analysis |
| 4. Ong *et al.,*2019^4^ | feasibility analysis |
| 5. Wang *et al.,*2019^5^ | feasibility analysis |
| 6. Lynch *et al.,*2020^6^ | feasibility analysis |
| 7. Parke *et al.,*2020^7^ | feasibility analysis |
| 8. Wang *et al.,*2020^8^ | feasibility analysis |
| 9. Bienvenu *et al.,*2021^9^ | feasibility analysis |
| 10. Georgescu *et al.,*2021^10^ | feasibility analysis |
| 11. Jawed *et al.,*2021^11^ | feasibility analysis |
| 12. Naef *et al.,*2021^12^ | feasibility analysis |
| 13. Droc *et al*.,2023^13^ | feasibility analysis |
| 14. Gai *et al*.,2021^14^ | feasibility analysis |
| 15. Blair *et al*.,2018^15^ | case report |
| 16. Esumi *et al*.,2020^16^ | case report |
| 17. Vlake *et al*.,2020^17^ | case report |
| 18. Kissel *et al*.,2021^18^ | case report |
| 19. Hill *et al*.,2022^19^ | case report |
| 20. Krogg *et al.,*2018^20^ | conference abstract |
| 21. Luetz *et al.,*2019^21^ | conference abstract |
| 22. Quah *et al*.,2019^22^ | conference abstract |
| 23. Suvajdzic *et al*.,2019^23^ | conference abstract |
| 24. Vlake *et al*.,2019^24^ | conference abstract |
| 25. Schecter.,2020^25^ | conference abstract |
| 26. Badke.,2022^26^ | discrepancy in outcome measures |
| 27. Bruno *al*.,2022^27^ | discrepancy in outcome measures |
| 28. Bruno *et al*.,2022^28^ | discrepancy in outcome measures |
| 29. Naef *et al*.,2023^29^ | discrepancy in outcome measures |
| 30. Raya.,2023^30^ | discrepancy in outcome measures |
| 31. Hereu *et al*.,2024^31^ | discrepancy in outcome measures |
| 32. Bravo *et al*.,2019^32^ | discrepancy in study subjects |
| 33. Spiegel *et al*.,2019^33^ | discrepancy in study subjects |
| 34. Ahmad *et al*.,2020^34^ | discrepancy in study subjects |
| 35. He *et al*.,2022^35^ | discrepancy in study subjects |
| 36. Vlake *et al*.,2022^36^ | discrepancy in study subjects |
| 37. Wu, *et al*.,2022^37^ | discrepancy in study subjects |
| 38. Bruder *et al*.,2024^38^  39. Rousseaux *et al*.,2020^39^ | discrepancy in study subjects  discrepancy in study subjects |
| 40. Dalir *et al*.,2024^40^  41. Ong *et al.*2020^41^ | discrepancy in study subjects  Quasi-experimental study (non-randomized controlled trial) |
| 42. Locke *et al*.,2024^42^ | Quasi-experimental study (non-randomized controlled trial) |

**R E F E R E N C E S**

1. Gerber SM, Jeitziner MM, Wyss P, et al. Visuo-acoustic stimulation that helps you to relax: a virtual reality setup for patients in the intensive care unit. Sci Rep. 2017;7(1):13228. doi:10.1038/s41598-017-13153-1

2. Turon M, Fernandez-Gonzalo S, Jodar M, et al. Feasibility and safety of virtual-reality-based early neurocognitive stimulation in critically ill patients. Ann Intensive Care. 2017;7(1):81. doi:10.1186/s13613-017-0303-4

3. Suvajdzic M, Bihorac A, Rashidi P, Ong T, Applebaum J. Virtual reality and human consciousness: the use of immersive environments in delirium therapy. Technoetic Arts. 2018;16(1):75-83. doi:10.1386/tear.16.1.75_1

4. Ong T, Ruppert M, Rashidi P, Ozrazgat-Baslanti T, Bihorac A, Suvajdzic M. The DREAMS project: improving the intensive care patient experience with virtual reality. Ithaca: Cornell University Library, arXiv.org; 2019. doi:10.48550/arxiv.1906.11706

5. Wang J, Zhang C, Jia Y, Shi C, Choi T, Xiao Q. Development of a virtual reality system for early mobilization of critically ill patients. Stud Health Technol Inform. 2019;264:1805-1806. doi:10.3233/SHTI190657

6. Wilding C, Young K, Cummins C, et al. Virtual reality to foster empathy in disability workers: a feasibility study during COVID-19. J Appl Res Intellect Disabil. 2023;36(1):132-142. doi:10.1111/jar.13042

7. Parke S, Hough CL, E BA. The feasibility and acceptability of virtual therapy environments for early ICU mobilization. Pm R. 2020;12(12):1214-1221. doi:10.1002/pmrj.12352

8. Jiani W, Chunyan Z, Yanrui J, Chenxi S, Qian X. Design and acceptability of virtual reality system for early mobilization of patients in a respiratory intensive care unit. Chinese Nursing Management. 2020;20(2):171-176. doi:10.3969/j.issn.1672-1756.2020.02.005

9. Bienvenu OJ. Posttraumatic stress phenomena in critical illness and intensive care survivors. Int Rev Psychiatry. 2021;33(8):691-698. doi:10.1080/09540261.2021.2017863

10. Georgescu RD, Dobrean A, Silaghi CA, Silaghi H. A virtual reality-based intervention for surgical patients: study protocol of a randomized controlled trial. Trials. 2021;22(1):289. doi:10.1186/s13063-021-05196-7

11. Jawed YT, Golovyan D, Lopez D, et al. Feasibility of a virtual reality intervention in the intensive care unit. Heart Lung. 2021;50(6):748-753. doi:10.1016/j.hrtlng.2021.05.007

12. Naef AC, Jeitziner MM, Gerber SM, et al. Virtual reality stimulation to reduce the incidence of delirium in critically ill patients: study protocol for a randomized clinical trial. Trials. 2021;22(1):174. doi:10.1186/s13063-021-05090-2

13. Droc G, Isac S, Nita E, et al. Postoperative cognitive impairment and pain perception after abdominal surgery-could immersive virtual reality bring more? A clinical approach. Medicina (Kaunas). 2023;59(11). doi:10.3390/medicina59112034

14. Yubiao G, Qingwei L, Minglei R, Jinze H, Xiaojing G, Lili W. The design and applicability evaluation of virtual reality-based rehabilitation system for ICU patients/. Chinese Journal of Emergency and Critical Care Nursing. 2023;4(10):892-897. doi:10.3761/j.issn.2096-7446.2023.10.005

15. Blair GJ, Kapil S, Cole SP, Rodriguez S. Virtual reality use in adult ICU to mitigate anxiety for a patient on v-v ECMO. J Clin Anesth. 2019;55:26-27. doi:10.1016/j.jclinane.2018.12.033

16. Esumi R, Yokochi A, Shimaoka M, Kawamoto E. Virtual reality as a non-pharmacologic analgesic for fasciotomy wound infections in acute compartment syndrome: a case report. J Med Case Rep. 2020;14(1):46. doi:10.1186/s13256-020-02370-4

17. Vlake JH, van Bommel J, Hellemons ME, Wils EJ, Gommers D, van Genderen ME. Intensive care unit-specific virtual reality for psychological recovery after ICU treatment for COVID-19; A brief case report. Front Med (Lausanne). 2020;7:629086. doi:10.3389/fmed.2020.629086

18. Kissel KA, Soo A, Bennett KT. Virtual reality as an adjunctive comfort measure in the intensive care and coronary care unit: a nurse-led quality improvement project. Pembroke: Canadian Association of Critical Care Nurses; 2021:5-13. doi:10.5737/23688653-324513

19. Hill JE, Twamley J, Breed H, et al. Scoping review of the use of virtual reality in intensive care units. Nurs Crit Care. 2022;27(6):756-771. doi:10.1111/nicc.12732

20. Krogg W, Golovyan D, Abshire L, et al. Development of a virtual reality environment to provide cognitive stimulation : a novel tool to reduce pain, anxiety, and delirium in critically ill patients. Am J Respir Crit Care Med. 2018;197

21. Luetz A, Grunow JJ, Morgeli R, et al. Innovative ICU solutions to prevent and reduce delirium and post-intensive care unit syndrome. Semin Respir Crit Care Med. 2019;40(5):673-686. doi:10.1055/s-0039-1698404

22. Mccleery JP, Zitter A, Solorzano R, et al. Safety and feasibility of an immersive virtual reality intervention program for teaching police interaction skills to adolescents and adults with autism. Autism Res. 2020;13(8):1418-1424. doi:10.1002/aur.2352

23. Suvajdzic M, Bihorac A, Rashidi P, et al. Developing a patient-centered virtual reality healthcare system to prevent the onset of delirium in ICU patients: IEEE; 2019:1-7. doi:10.1109/SeGAH.2019.8882442

24. Wheeler A, Bloch E, Blaylock S, et al. Delirium education for family caregivers of patients in the intensive care unit: a pilot study. PEC Innov. 2023;2:100156. doi:10.1016/j.pecinn.2023.100156

25. Stollings JL, Kotfis K, Chanques G, Pun BT, Pandharipande PP, Ely EW. Delirium in critical illness: clinical manifestations, outcomes, and management. Intensive Care Med. 2021;47(10):1089-1103. doi:10.1007/s00134-021-06503-1

26. Badke CM, Krogh-Jespersen S, Flynn RM, Shukla A, Essner BS, Malakooti MR. Virtual reality in the pediatric intensive care unit: patient emotional and physiologic responses. Front Digit Health. 2022;4:867961. doi:10.3389/fdgth.2022.867961

27. Bruno RR, Bruining N, Jung C. Virtual reality in intensive care. Intensive Care Med. 2022;48(9):1227-1229. doi:10.1007/s00134-022-06792-0

28. Bruno RR, Wolff G, Wernly B, et al. Virtual and augmented reality in critical care medicine: the patient's, clinician's, and researcher's perspective. Crit Care. 2022;26(1):326. doi:10.1186/s13054-022-04202-x

29. Naef AC, Gerber SM, Single M, et al. Effects of immersive virtual reality on sensory overload in a random sample of critically ill patients. Front Med (Lausanne). 2023;10:1268659. doi:10.3389/fmed.2023.1268659

30. Raya L, Ruiz JJ, Fabian M, et al. Development of a virtual reality tool for the treatment of pediatric patients in the ICU. In: Mike P ed. United States: IEEE; 2023:69-77. doi:10.1109/MCG.2023.3239676

31. Martí-Hereu L, Navarra-Ventura G, Navas-Pérez AM, et al. Usage of immersive virtual reality as a relaxation method in an intensive care unit. Enfermería Intensiva (English Ed.). 2024;35(2):107-113. doi:10.1016/j.enfie.2023.08.005

32. Garcia-Bravo S, Cuesta-Gomez A, Campuzano-Ruiz R, et al. Virtual reality and video games in cardiac rehabilitation programs. A systematic review. Disabil Rehabil. 2021;43(4):448-457. doi:10.1080/09638288.2019.1631892

33. Spiegel B, Fuller G, Lopez M, et al. Virtual reality for management of pain in hospitalized patients: a randomized comparative effectiveness trial. PLoS One. 2019;14(8):e0219115. doi:10.1371/journal.pone.0219115

34. Ahmed N, Mauad V, Gomez-Rojas O, et al. The impact of rehabilitation-oriented virtual reality device in patients with ischemic stroke in the early subacute recovery phase: study protocol for a phase III, single-blinded, randomized, controlled clinical trial. J Cent Nerv Syst Dis. 2020;12:1179573519899471. doi:10.1177/1179573519899471

35. He M, Li X, Zhang T, Jin X, Hu C. The fifth generation mobile communication technology plus virtual reality system for intensive care unit visits during COVID-19 pandemic: keep the delirium away. J Nurs Manag. 2022;30(8):3885-3887. doi:10.1111/jonm.13450

36. Vlake JH, van Bommel J, Wils EJ, et al. Intensive care unit-specific virtual reality for critically ill patients with COVID-19: multicenter randomized controlled trial. J Med Internet Res. 2022;24(1):e32368. doi:10.2196/32368

37. Wu Y, Chen J, Ma W, Guo L, Feng H. Virtual reality in preoperative preparation of children undergoing general anesthesia: a randomized controlled study. Anaesthesiologie. 2022;71(Suppl 2):204-211. doi:10.1007/s00101-022-01177-w

38. Bruder AL, Gururaja A, Narayani N, Kleinpell R, Schlesinger JJ. Patients' perceptions of virtual live music in the intensive care unit. Am J Crit Care. 2024;33(1):54-59. doi:10.4037/ajcc2024140

39. Rousseaux F, Faymonville ME, Nyssen AS, et al. Can hypnosis and virtual reality reduce anxiety, pain and fatigue among patients who undergo cardiac surgery: a randomised controlled trial. Trials. 2020;21(1):330. doi:10.1186/s13063-020-4222-6

40. Dalir Z, Seddighi F, Esmaily H, Abbasi TM, Ramezanzade TE. Effects of virtual reality on chest tube removal pain management in patients undergoing coronary artery bypass grafting: a randomized clinical trial. Sci Rep. 2024;14(1):2918. doi:10.1038/s41598-024-53544-9

41. Ong TL, Ruppert MM, Akbar M, et al. Improving the intensive care patient experience with virtual reality-a feasibility study. Crit Care Explor. 2020;2(6):e0122. doi:10.1097/CCE.0000000000000122

42. Locke BW, Tsai TY, Reategui-Rivera CM, Gabriel AS, Smiley A, Finkelstein J. Immersive virtual reality use in medical intensive care: mixed methods feasibility study. JMIR Serious Games. 2024;12:e62842. doi:10.2196/62842
