# Supplementary material for: Effectiveness of Virtual Reality–Based Early Rehabilitation Strategies on Pain, Sleep, Anxiety, Balance, Cognition, and Limb Motor Function in Adult Intensive Care Unit Patients: Systematic Review and Meta-Analysis of Randomized Controlled Trials
Source: J Med Internet Res. 2026 Mar 6;28:e81865. doi: 10.2196/81865 (PMC12978899; doi:10.2196/81865)
Supplement: Multimedia Appendix 3 [file jmir-v28-e81865-s003.docx]

**Table 1. Characteristics of studies identified from the literature search.**

| Study  (Author,  year) | Country | Types of Diseases | Timing of Intervention Initiation | Study Period | VR Device Type | Content Type | Sample size  (Intervention /Control) | Age | Intervention | | Duration and frequency of interventions | Outcome indicator | ICU length of stay（day） | Adverse Events |
| --- | --- | --- | --- | --- | --- | --- | --- | --- | --- | --- | --- | --- | --- | --- |
|  |  |  |  |  |  |  |  | **Intervention/ Control** | **Intervention**  **group** | **Control group** |  |  |  |  |
| Caballero,2025 | Brazil | Patients admitted to the ICU with acute decompensated heart failure (ADHF) | 24 hours after ICU admission | Jan 2023 - Jan 2024 | Fully Immersive HMD | Early functional mobility rehabilitation | 23/24 | 62.0±11.2/ 57.4±12.8 | Participants received up to 3 standard early mobilization sessions using immersive VR headset displaying interactive 360° videos to explore virtual environments. | Participants received up to 3 standard early mobilization sessions in the ICU, including bedside cycling in sitting position, upright position training, and ambulation. | 10-20 minutes per session, adjusted based on patient tolerance. | ①② | Not Reported | Only 2 of 60 patients were excluded due to "clinical deterioration" without any mobilization sessions. No adverse events leading to withdrawal were attributed to the intervention itself. The Net Promoter Score showed 78.3% were "promoters". |
| Kim,2025 | South Korea | Patients with routine general surgical ICU admission | Day of ICU admission | Apr - Sep 2020 | Fully Immersive HMD | VR meditation for sleep promotion | 49/47 | 67.63 ± 11.31/  66.28 ± 12.5 | Patients received standard sleep intervention along with a 20-minute VR meditation session of their choice, which included nature environments, focused meditation, breathing exercises, and tree mindfulness practices. | Routine care (including noise and light reduction, with provision of eye masks and earplugs as required) | Nightly 20-minute interventions at 9:00 PM, continuing until ICU discharge | ③④⑤ | Intervention:  2.28 ± 1.23  Control:  2.36 ± 1.45 | Not Reported |
| Laghlam,2021 | French | Undergo drain removal after cardiac surgery | Second day after cardiac surgery | Sep 2019 - Jul 2020 | Fully Immersive HMD | VR relaxation therapy for pain relief | 90/90 | 68.0±10/  68±10.96 | Patients experience a choice of five different immersive environments (360° video): snow-capped mountains, landscapes of India or the French Camargue, a balloon ride, or a canoe descent. | Active analgesia using an equimolar mixture of oxygen and nitrous oxide (Kalinox®). | 5 min before 10 min after drain removal, 24 weeks of intervention. | ⑥⑦ | Not Reported | Three minor side effects were declared in the VR group (2 vertigo and 1 nausea) and also three in the Kalinox® group (2 euphoria and 1 headache). |
| Lee, 2020 | South Korea | Cardiovascular diseases (angina, myocardial infarction, heart failure, and cardiomyopathy) | Day of ICU admission | Dec 2018 - Mar 2019 | Fully Immersive HMD | VR meditation for sleep promotion | 24/24 | 69.46 ± 16.27/  63.38 ± 16.53 | The patient wears a head-mounted monitor to autonomously select a meditation video that includes nature scenes such as mountains, oceans, or lakes while background sounds and calming music play. | Routine sleep hygiene protocol (including provision of eye masks/earplugs upon request) | 30-minute intervention period prior to bedtime on ICU admission day | ④⑧ | Mean: 2.79 days | Not Reported |
| Li, 2024 | China | Acute myocardial infarction  Cardiac ICU | Day of ICU admission | Dec 2022 - Feb 2023 | Fully Immersive HMD | Early cognitive rehabilitation training | 70/78 | 58.3 ±9.3/ 59.9±9.5 | Participants wore head-mounted displays hypnotherapy (day 1), positive cognition (days 2 and 3), music therapy (days 4 and 5) and relaxation therapy (days 6 and 7). Each module contains several scenarios from which participants can choose 1-2 each day. | Mental health support (Mental health support, including brief mental health-related education and daily mental health care provided by nurses.) | Approximately 30 minutes of intervention per day for a total of 7 days. | ⑨⑩ ⑪⑫⑬⑭ | Not Reported | 5 cases of cybersickness reported (3 discomfort from HMD, 1 dizziness, 1 headache). No participant dropped out. No serious adverse events. |
| Merliot,2022 | French | Patients in the combined medical-surgical ICU | Day of ICU admission | Jul - Dec 2019 | Fully Immersive HMD | VR relaxation therapy for stress reduction | 56/54 | -/- | Patients were able to use VR goggles to watch real movies, perform breathing exercises, and listen to hypnotic voice guidance. | A 15-minute period for relaxation and distraction, utilizing methods such as television or radio, during which no medical care procedures are to be performed. | Administered once, with each session lasting approximately 15 minutes | ⑥⑦ | 8.0 [5.2–12.1] | 3 patients reported events (claustrophobia, agitation, dyspnea). 6% of sessions were interrupted early due to visual problems, anxiety, or care interruptions. |
| Mo, 2021 | China | ICU patients with multiple fractures | Day of ICU admission | Jun 2019 - Jun 2020 | Fully Immersive HMD | Early motor rehabilitation | 46/46 | 40.85±4.19/ 40.54±4.32 | VR system with sensors projected patient's movements into virtual scenarios (e.g., running, dodging) for guided exercises. | Multidisciplinary rehabilitation care: education, infection control, personalized plans, nutritional support. | 30-45 min/session, once daily, 5 times/week for 3 months. | ⑮⑯⑰⑱ | Not Reported | Not Reported |
| Ventura, 2021 | Spanish | Patients in the combined medical-surgical ICU | After stabilization on ICU admission day | Nov 2015 - Sep 2020 | Non-Immersive (Desktop) | Early cognitive rehabilitation training | 21/21 | 69.1±37.19/  67.7±36.07 | The patient is placed in a relaxation environment (tropical island) consisting of 4 scenes with realistic natural sounds (wheat field, beach, forest, and mountain landscape), where the patient can walk accompanied by a virtual avatar, who promptly orientates the patient and provides instructions to encourage cognitive exercises and relaxation. | Conventional cognitive exercises (i.e., exercises where patients lift each arm against gravity for cognitive interaction training). | Minimum two interventions of 15-20 min each during ICU care, with follow-up after 1 month. | ⑲⑳㉑㉒ | Intervention:  16 [6–76]  Control:  10 [5–73] | 13.2% of sessions stopped due to fatigue (50%), drowsiness, dizziness, anxiety, confusion. No adverse events. |
| Ouyang,2022 | China | ICU patients aged ≥60 years | Day of ICU admission | Sep 2021 - Jan 2022 | Fully Immersive HMD | Early cognitive rehabilitation training | 23/24 | 67.48±5.03/ 69.38±5.19 | Patients viewed 360° nature scenes (e.g., mountains, rivers) with corresponding sounds or light music via HMD. | Routine ICU care (vital signs monitoring, diet, medication, activity guidance). | 15 min/session, twice daily for 1 week. | ⑳㉑㉓㉔ | 7(7,8)  7(7,8) | 7 patients had mild dizziness (16 episodes), 4 had mild nausea (4 episodes). Symptoms resolved within 5 mins of rest. No serious adverse events. |
| Wang,2025 | China | RICU patients with severe respiratory diseases (≥18 years) | Upon stabilization after RICU admission | Mar 2019 - Jan 2020 | Fully Immersive HMD | Early functional mobility rehabilitation | 33/35 | 61±8.66/ 56±8.37 | System used human-computer interaction for active limb movements (e.g., virtual piano, games for wrist/shoulder/elbow exercises). | Standard rehabilitation exercises (passive limb movements, assisted standing, ambulation) under supervision. | 20 min/session, 3 times daily (morning, noon, evening) for at least 3 days. | ㉕㉖㉗㉘㉙㉚㉛ | Not Reported | Vital signs fluctuated within ±10% during exercise. No exercise-related adverse reactions or safety events were observed. |
| Weng, 2023 | China | Patients undergoing tumor surgery, including those with esophageal cancer, gastric cancer, ovarian cancer, brain tumors, and lymphoma | >6 hours after ICU admission | Oct 2022 - Oct 2023 | Fully Immersive HMD | Early delirium prevention | 42/42 | 59.13±5.21/  58.62±5.18 | Provide an immersive audio-visual experience, listen to or watch music, episodes, etc.; hear and see family members record videos, simulate family moments, but also access to the virtual seabed, forest or countryside scenery, etc.; play memory games, intellectual challenges, and other cognitive stimulation. | Routine care（including scheduled turning, intermittent pneumatic compression therapy, endotracheal suctioning, and twice-daily active-assisted/passive range-of-motion rehabilitation exercises.） | Twice daily throughout the entire ICU admission | ㉜㉝㉞ | Not Reported | Not Reported |
| Xia,2023 | China | Patients with routine ICU admission | 24 hours after ICU admission | Nov 2021 - Jan 2022 | Fully Immersive HMD | Early motor rehabilitation | 37/31 | 62.46±13.95/  62.58±14.74 | Utilize VR technology to simulate interactive scenarios including bird-catching, piano-playing, block-building, as well as calisthenics in a virtual park and tennis matches, enabling patients to complete corresponding exercise tasks. | Routine early mobilization protocol involves daily exercise sessions at 10:00 AM, comprising pre-activity preparation and a 6-section rehabilitation routine | Once daily at 10:00 AM, spanning from 24 hours post-ICU admission until ICU discharge | ㉕㉙㉚㉟ | Intervention:  4.65±2.82 Control:  6.90±2.40 | 1 patient in VR group had significant BP drop, intervention stopped and managed. 1 in control group had leg pain, intervention paused. No other risks like falls. |
| Yesilot, 2022 | Turkish | Post-laparoscopic sleeve gastrectomy | 6 hours after ICU admission | Sep - Dec 2019 | Fully Immersive HMD | VR relaxation therapy for pain relief | 55/55 | 36.62 ± 9.65/  37.35 ± 12.35 | Lie back and watch virtual videos that include images of nature, such as mountains, seas and forests, accompanied by relaxing music. | The patient received pharmacological treatment and remained supine in bed for rest. | A single intervention lasting 30 minutes. | ㊱㊲ | Not Reported | Not Reported |
| Yan,2024 | China | Cardiac surgery patients | Upon stabilization on ICU admission day | Jan - May 2024 | Fully Immersive HMD | Preoperative visit, VR relaxation therapy | 39/41 | 59.26±10.01/ 61.00±10.71 | Using VR to introduce ICU environmental information, ICU healthcare team composition and nurse-to-patient ratios, postoperative catheter/tube management, potential postoperative discomforts and coping strategies, respiratory function training, sleep health guidance, postoperative activity and rehabilitation instructions, and family support. | Routine care: Written materials supplemented by verbal explanation of medical risks, anesthesia methods, respiratory function recovery training, key points of catheter/tube care, and early rehabilitation exercise protocols. | A single intervention | ㉜㉝ | Not Reported | Not Reported |
| Zeng,2021 | China | Postoperative patients with traumatic brain injuries | Day of ICU admission | Jan 2019 - Dec 2020 | Fully Immersive HMD | Early cognitive and motor rehabilitation | 38/38 | 48.75 ± 4.19/  48.52 ±4.36 | Patients interact within a virtual environment by moving their upper limbs to catch falling coconuts, accurately avoiding obstacles, and classifying/assembling various household items displayed on the screen. | Routine cognitive-behavioral intervention, encompassing cognitive restructuring, behavioral strategies, and physical functional exercises. | 5 days per week | ⑮㊳ | Not Reported | Not Reported |
| Zhu,2023 | China | Postoperative patients in the intensive care unit following liver transplantation. | Upon stabilization on ICU admission day | Jan 2020 - Oct 2022 | Fully Immersive HMD | Early functional mobility rehabilitation | 30/30 | 57.33±4.39/  57.91±4.26 | Early-stage bedridden rehabilitation employs low-energy expenditure programs, including simulated penguin-tapping, fruit-slicing, and remote-controlled car racing games; later-stage programs focus on enhancing muscular endurance and active motor control through simulated table tennis, skiing, and other dynamic exercises. | Standard care and rehabilitation training encompass routine health education, dietary guidance, respiratory exercises, bedside defecation/cough training, motor exercises, scheduled repositioning, and pain management strategies. | Once daily for a total of 2 weeks. | ⑮㊴㊵ | Not Reported | Intervention was to be stopped if nausea or dizziness occurred. No specific adverse events reported. |

Note: ① Feedback and suggestions (Qualitative interview)；② Degree of recommendation: Likert scale；③ Subjective sleep (The Korean version of Verran and Snyder-Halpern Sleep Scale (VSHSS))；④ Objective sleep: Smart bracelet Fitbit Charge 3 (Fitbit Inc., San Francisco, CA, USA)；⑤ Delirium (The Korean version of CAM-ICU)；⑥ Pain (Numeric Rating Scale (NRS))；⑦ Anxiety (Numeric Rating Scale (NRS))；⑧ Subjective sleep (Korean Sleep Scale A)；⑨ Anxiety (HAM-A scores, State-Trait Anxiety Inventory (STAI))；⑩ Cognition (The Perceived Deficits Questionnaire for Depression (PDQ-D))；⑪ Somatic symptoms (PHQ-15, Patient Health Questionnaire-15)；⑫ Perceived well-being (SF-12 MCS, 12-Item Short Form Health Survey Mental Component Score)；⑬ Subjective sleep (Pittsburgh Sleep Quality Index (PSQI))；⑭ Quality of life (12-Item Short Form Health Survey Scale (SF-12))；⑮ Balance function (Berg Balance Scale (BBS))；⑯ Motor function (Fugl-Meyer Assessment (FMA))；⑰ Walking ability (Functional Ambulation Category Scale (FAC))；⑱ Exercise adherence: Self-developed assessment tool；⑲ Cognition (14 neuropsychological measures)；⑳ Anxiety (Hospital Anxiety and Depression Scale (HADS))；㉑ Depression (Hospital Anxiety and Depression Scale (HADS))；㉒ Post-traumatic stress disorder (Davidson Trauma Scale (DTS))；㉓ Cognitive function status (Montreal Cognitive Assessment (MoCA))；㉔ Subjective sleep (Richards-Campbell Sleep Questionnaire (RCSQ), Pittsburgh Sleep Quality Index (PSQI))；㉕ Daily total exercise time (minutes; min)；㉖ Average daily out-of-bed exercise time (minutes; min)；㉗ Exercise adherence: Self-developed assessment tool；㉘ Muscle strength (MRC score)；㉙ Grip strength (Dynamometer)；㉚ ICU length of stay (days; d)；㉛ Mechanical ventilation time (days; d)；㉜ Anxiety (Self-Rating Anxiety Scale (SAS))；㉝ Subjective sleep (Richards-Campbell Sleep Questionnaire (RCSQ))；㉞ Delirium severity (Confusion Assessment Method (CAM))；㉟ ICU weakness (Medical Research Council Score (MRC))；㊱ Pain (Numeric Pain Rating Scale (NPRS))；㊲ Anxiety (Faces Anxiety Scale)；㊳ Cognitive function status (Montreal Cognitive Assessment and MMSE)；㊴ Muscle strength (Manual Muscle Testing (MMT))；㊵ Postoperative recovery quality (Postoperative Quality Recovery Scale (PQRS)).
