# Supplementary material for: Effectiveness of Virtual Reality–Based Early Rehabilitation Strategies on Pain, Sleep, Anxiety, Balance, Cognition, and Limb Motor Function in Adult Intensive Care Unit Patients: Systematic Review and Meta-Analysis of Randomized Controlled Trials
Source: J Med Internet Res. 2026 Mar 6;28:e81865. doi: 10.2196/81865 (PMC12978899; doi:10.2196/81865)
Supplement: Multimedia Appendix 5 [file jmir-v28-e81865-s005.docx]

**Summary of Findings (SoF) Table: Virtual Reality-Based Early Rehabilitation vs. Standard Care in the ICU**

| **Virtual Reality-based Early Rehabilitation Intervention compared to standard early rehabilitation for critically ill patients in the intensive care unit.** | | | | | |
| --- | --- | --- | --- | --- | --- |
| **Bibliography:** Wu F, Wu Y, Xing Y, Zhang R, Cai W. Effectiveness of Virtual Reality-Based Early Rehabilitation Strategies on Pain, Sleep, Anxiety, Balance, Cognition, and Limb Motor Function in Adult ICU Patients: A Systematic Review and Meta-Analysis of Randomized Controlled Trials. J Med Internet Res. Forthcoming 2025. | | | | | |
| **Outcomes** | **No of Participants (studies)** Follow up | **Quality of the evidence** (GRADE) | **Relative effect (95% CI)** | **Anticipated absolute effects** | |
|  |  |  |  | *Time frame is Activities were initiated within 72 hours of ICU admission.* | |
|  |  |  |  | **Risk with Routine care/Non-VR-based intervention** | **Risk difference with Virtual Reality-based early rehabilitation intervention** (95% CI) |
| **Anxiety** | 801 (8 studies) | ⊕⊝⊝⊝ **VERY LOW**^1,2,3,4^ due to risk of bias, inconsistency, imprecision, publication bias | SMD -0.86 (-1.85 to 0.13) | The mean anxiety score in the control groups was -3.08 points. | The mean anxiety in the intervention groups was **0.86 standard deviations lower** (1.85 lower to 0.13 higher). |
| **Subjective sleep quality** | 500 (6 studies) | ⊕⊝⊝⊝ **VERY LOW**^5,6,7^ due to risk of bias, inconsistency, imprecision | SMD 3.36 (0.77 to 5.94) | The mean subjective sleep quality score in the control groups was 15.70 points. | The mean subjective sleep quality in the intervention groups was **3.36 standard deviations higher** (0.77 higher to 5.94 higher). |
| **Objective total sleep time** | 141 (2 studies) | ⊕⊕⊕⊝ **MODERATE**^8^ due to risk of bias | SMD 0.08 (-0.07 to 0.22) | The mean total sleep time in the control groups was 337.60 minutes. | The mean total sleep time in the intervention groups **was 0.08 standard deviations higher** (0.07 lower to 0.22 higher). |
| **Objective wake after sleep onset** | 141 (2 studies) | ⊕⊝⊝⊝ **VERY LOW**^8,9,10^ due to risk of bias, inconsistency, imprecision | SMD -0.42 (-5.85 to 5.00) | The mean wake after sleep onset in the control groups was 59.00 minutes. | The mean wake after sleep onset in the intervention groups was **0.42 standard deviations lower** (5.85 lower to 5.00 higher). |
| **Objective sleep efficiency** | 141 (2 studies) | ⊕⊝⊝⊝ **VERY LOW**^8,11,12^ due to risk of bias, inconsistency, imprecision | SMD 0.24 (-5.21 to 5.68) | The mean sleep efficiency in the control groups was 82.98%. | The mean sleep efficiency in the intervention groups was **0.24 standard deviations higher** (5.21 lower to 5.68 higher). |
| **Pain** | 220 (2 studies) | ⊕⊕⊝⊝ **LOW**^13,14^ due to risk of bias, imprecision | SMD -0.58 (-2.58 to 1.43) | The mean pain score in the control groups was -1.10 points. | The mean pain score in the intervention groups was 0.58 **standard deviations lower** (2.58 lower to 1.43 higher). |
| **Cognitive function** | 271 (3 studies) | ⊕⊕⊝⊝ **LOW**^16,17^ due to risk of bias, imprecision | SMD 0.78 (0.16 to 1.39) | The mean cognitive function score in the control groups was 1.84 points. | The mean cognitive function in the intervention groups was **0.78 standard deviations higher** (0.16 higher to 1.39 higher). |
| **Balance ability** | 228 (3 studies) | ⊕⊕⊕⊝ **MODERATE**^15^ due to risk of bias | SMD 0.97 (0.74 to 1.20) | The mean balance score in the control groups was 6.7 points. | The mean balance ability at short-term follow-up in the intervention groups was **0.97 standard deviations higher** (0.74 higher to 1.20 higher) |
| **Limb motor function** | 168 (2 studies) | ⊕⊕⊝⊝ **LOW**^18,19^ due to risk of bias, imprecision | SMD 1.40 (-0.23 to 3.02) | The mean limb motor function score in the control groups was 3.20 points. | The mean limb motor function in the intervention groups was **1.40 standard deviations higher** (0.23 lower to 3.02 higher). |
| *The basis for the **assumed risk** (e.g. the median control group risk across studies) is provided in footnotes. The **corresponding risk** (and its 95% confidence interval) is based on the assumed risk in the comparison group and the **relative effect** of the intervention (and its 95% CI).  The risk difference expresses the intervention effect as a Standardized Mean Difference (SMD). An SMD of 0.2 represents a small, 0.5 a moderate, and 0.8 a large difference. **CI:** Confidence interval; **SMD**: Standardized Mean Difference. | | | | | |
| GRADE Working Group grades of evidence **High quality:** Further research is very unlikely to change our confidence in the estimate of effect.  **Moderate quality:** Further research is likely to have an important impact on our confidence in the estimate of effect and may change the estimate. **Low quality:** Further research is very likely to have an important impact on our confidence in the estimate of effect and is likely to change the estimate. **Very low quality:** We are very uncertain about the estimate. | | | | | |
| ^1^ Among the 8 studies, 5 were rated as high risk of bias and 3 had some concerns. This means that over half of the studies had a high risk of bias that could seriously affect the credibility of the results (e.g., lack of allocation concealment, no blinding, high attrition rate, etc.). ^2^ There was extremely high heterogeneity among the studies (I² = 95.4%, P < 0.01), indicating that the differences in effect sizes between studies far exceeded what could be explained by random error.  ^3^ The 95% CI of the pooled effect (-1.85 to 0.13) included both "no effect" (0) and "clinically important benefit" (a negative SMD with a large absolute value).  ^4^ Although the statistical evidence is inconsistent and of limited power, our preliminary analysis suggests there may be a risk of publication bias.  ^5^ Among the 6 included RCTs, 4 were assessed as "high risk of bias" and 2 as "having some concerns." This means that over half of the studies had serious methodological flaws (e.g., inadequate allocation concealment, poor implementation of blinding), which are likely to lead to an overestimation of the intervention effect.  ^6^ The statistical heterogeneity was extremely high (I² = 97.5%, P < .01), indicating that the differences in effect sizes between studies far exceeded what could be explained by random error.  ^7^ The 95% confidence interval for the pooled effect SMD=3.36 was very wide (0.77 to 5.94), spanning from a "moderate effect" to an "extremely large effect," indicating very imprecise estimation of the effect size. ^8^ Among the 2 included RCTs, 1 was "high risk of bias" and 1 had "some concerns." This means 50% of the studies had serious methodological flaws. With a very small total number of studies, a single high-risk study can have a major impact on the overall reliability of the evidence, constituting what GRADE considers a "serious limitation."  ^9^ The statistical heterogeneity was high (I² = 81.6%), and the test was statistically significant (P = 0.0199), indicating that the differences in effect sizes between studies far exceeded what could be explained by random error.  ^10^ The 95% confidence interval for the pooled effect SMD = -0.42 was extremely wide (-5.85 to 5.00), with a width exceeding 10 standard deviation units.  ^11^ The statistical heterogeneity was high (I² = 82.1%), and the test was statistically significant (P = 0.0181), indicating that the differences in effect sizes between studies were too large to be explained by random error.  ^12^The 95% confidence interval for the pooled effect SMD = 0.24 was -5.21 to 5.68. The width of this interval (approximately 11 standard deviation units) is extremely abnormal.  ^13^ Among the 3 RCTs included in the final analysis, 1 was "high risk of bias" and 2 had "some concerns." This means all studies had some methodological flaws, with one-third having serious flaws. ^14^ The 95% confidence interval for the pooled effect SMD = -0.58 was very wide (-2.58 to 1.43) and included the line of no effect (0).  ^15^ All 3 included RCTs were rated as "high risk of bias." This means all studies had serious methodological flaws (e.g., inadequate allocation concealment, no blinding of patients or outcome assessors, etc.).  ^16^ All 3 included studies were rated as "high risk of bias." This means the studies had serious methodological flaws that could systematically overestimate or underestimate the intervention effect, significantly reducing our confidence in the effect estimate. ^17^ The 95% confidence interval for the pooled effect SMD=0.61 was relatively wide (-0.05 to 1.27) and included the line of no effect (0).  ^18^ Both included RCTs were rated as "high risk of bias." With a very limited evidence base, the fact that all studies had serious methodological flaws fundamentally questions the truthfulness of the effect estimate and significantly reduces confidence.  ^19^ The 95% confidence interval for the pooled effect SMD = 1.40 was extremely wide (-0.23 to 3.02) and included the line of no effect (0). | | | | | |

**GRADE Evidence Profile: Virtual Reality-Based Early Rehabilitation vs. Routine Care in the ICU**

| **Question: Should Virtual Reality-based early rehabilitation intervention vs Routine care/Non-VR-based intervention be used in Adults admitted to the intensive care unit.? Bibliography: Wu F, Wu Y, Xing Y, Zhang R, Cai W. Effectiveness of Virtual Reality-Based Early Rehabilitation Strategies on Pain, Sleep, Anxiety, Balance, Cognition, and Limb Motor Function in Adult ICU Patients: A Systematic Review and Meta-Analysis of Randomized Controlled Trials. J Med Internet Res. Forthcoming 2025.** | | | | | | | | | | | |
| --- | --- | --- | --- | --- | --- | --- | --- | --- | --- | --- | --- |
| **Quality assessment** | | | | | | | **Summary of Findings** | | | | |
| **Participants (studies) Follow up** | **Risk of bias** | **Inconsistency** | **Indirectness** | **Imprecision** | **Publication bias** | **Overall quality of evidence** | **Study event rates (%)** | | **Relative effect** (95% CI) | **Anticipated absolute effects** *Time frame is Activities were initiated within 72 hours of ICU admission.* | |
|  |  |  |  |  |  |  | **With Routine care/Non-VR-based intervention** | **With Virtual Reality-based early rehabilitation intervention** |  | **Risk with Routine care/Non-VR-based intervention** | **Risk difference with Virtual Reality-based early rehabilitation intervention** (95% CI) |
| **Anxiety** (CRITICAL OUTCOME; Better indicated by lower values) | | | | | | | | | | | |
| 801 (8 studies) | serious^1^ | very serious^2^ | no serious indirectness | serious^3^ | reporting bias strongly suspected^4^ | ⊕⊝⊝⊝ **VERY LOW**^1,2,3,4^ due to risk of bias, inconsistency, imprecision, publication bias | 405 | 396 | SMD -0.86 (-1.85 to 0.13) | The mean anxiety score in the control groups was -3.08 points. | The mean anxiety in the intervention groups was **0.86 standard deviations lower** (1.85 lower to 0.13 higher) |
| **Subjective sleep quality** (CRITICAL OUTCOME; Better indicated by lower values) | | | | | | | | | | | |
| 500 (6 studies) | serious^5^ | serious^6^ | no serious indirectness | serious^7^ | undetected | ⊕⊝⊝⊝ **VERY LOW**^5,6,7^ due to risk of bias, inconsistency, imprecision | 246 | 254 | SMD 3.36 (0.77 to 5.94) | The mean subjective sleep quality score in the control groups was 15.70 points. | The mean subjective sleep quality in the intervention groups was **3.36 standard deviations higher** (0.77 higher to 5.94 higher). |
| **Objective total sleep time** (Better indicated by lower values) | | | | | | | | | | | |
| 141 (2 studies) | serious^8^ | no serious inconsistency | no serious indirectness | no serious imprecision | undetected | ⊕⊕⊕⊝ **MODERATE**^8^ due to risk of bias | 69 | 72 | SMD 0.08 (-0.07 to 0.22) | The mean total sleep time in the control groups was 337.60 minutes. | The mean total sleep time in the intervention groups **was 0.08 standard deviations higher** (0.07 lower to 0.22 higher). |
| **Objective wake after sleep onset** (Better indicated by lower values) | | | | | | | | | | | |
| 141 (2 studies) | serious^8^ | serious^9^ | no serious indirectness | Serious^10^ | undetected | ⊕⊝⊝⊝ **VERY LOW**^8,9,10^ due to risk of bias, inconsistency, imprecision | 69 | 72 | SMD -0.42 (-5.85 to 5.00) | The mean wake after sleep onset in the control groups was 59.00 minutes. | The mean wake after sleep onset in the intervention groups was **0.42 standard deviations lower** (5.85 lower to 5.00 higher). |
| **Objective sleep efficiency** (Better indicated by lower values) | | | | | | | | | | | |
| 141 (2 studies) | serious^8^ | serious^11^ | no serious indirectness | very serious^12^ | undetected | ⊕⊝⊝⊝ **VERY LOW**^8,11,12^ due to risk of bias, inconsistency, imprecision | 69 | 72 | SMD 0.24 (-5.21 to 5.68) | The mean sleep efficiency in the control groups was 82.98%. | The mean sleep efficiency in the intervention groups was **0.24 standard deviations higher** (5.21 lower to 5.68 higher). |
| **Pain** (CRITICAL OUTCOME; Better indicated by lower values) | | | | | | | | | | | |
| 220 (2 studies) | serious^13^ | no serious inconsistency | no serious indirectness | serious^14^ | undetected | ⊕⊕⊝⊝ **LOW**^13,14^ due to risk of bias, imprecision | 111 | 109 | SMD -0.58 (-2.58 to 1.43) | The mean pain score in the control groups was -1.10 points. | The mean pain score in the intervention groups was **0.58 standard deviations lower** (2.58 lower to 1.43 higher). |
| **Cognitive function** (Better indicated by lower values) | | | | | | | | | | | |
| 271 (3 studies) | serious^16^ | no serious inconsistency | no serious indirectness | serious^17^ | undetected | ⊕⊕⊝⊝ **LOW**^16,17^ due to risk of bias, imprecision | 132 | 139 | SMD 0.78 (0.16 to 1.39) | The mean cognitive function score in the control groups was 1.84 points. | The mean cognitive function in the intervention groups was **0.78 standard deviations higher** (0.16 higher to 1.39 higher). |
| **Balance ability** (CRITICAL OUTCOME;Better indicated by lower values) | | | | | | | | | | | |
| 228 (3 studies) | serious^15^ | no serious inconsistency | no serious indirectness | no serious imprecision | undetected | ⊕⊕⊕⊝ **MODERATE**^15^ | 114 | 114 | SMD 0.97 (0.74 to 1.20) | The mean balance score in the control groups was 6.7 points. | The mean balance ability at short-term follow-up in the intervention groups was **0.97 standard deviations higher** (0.74 higher to 1.20 higher) |
| **Limb motor function** (CRITICAL OUTCOME;Better indicated by lower values) | | | | | | | | | | | |
| 168 (2 studies) | serious^18^ | no serious inconsistency | no serious indirectness | serious^19^ | undetected | ⊕⊕⊝⊝ **LOW**^18,19^ due to risk of bias, imprecision | 84 | 84 | SMD 1.40 (-0.23 to 3.02) | The mean limb motor function score in the control groups was 3.20 points. | The mean limb motor function in the intervention groups was **1.40 standard deviations higher** (0.23 lower to 3.02 higher). |

^1^ Among the 8 studies, 5 were rated as high risk of bias and 3 had some concerns. This means that over half of the studies had a high risk of bias that could seriously affect the credibility of the results (e.g., lack of allocation concealment, no blinding, high attrition rate, etc.).
^2^ There was extremely high heterogeneity among the studies (I² = 95.4%, P < 0.01), indicating that the differences in effect sizes between studies far exceeded what could be explained by random error.

^3^ The 95% CI of the pooled effect (-1.85 to 0.13) included both "no effect" (0) and "clinically important benefit" (a negative SMD with a large absolute value).

^4^ Although the statistical evidence is inconsistent and of limited power, our preliminary analysis suggests there may be a risk of publication bias.
^5^ Among the 6 included RCTs, 4 were assessed as "high risk of bias" and 2 as "having some concerns." This means that over half of the studies had serious methodological flaws (e.g., inadequate allocation concealment, poor implementation of blinding), which are likely to lead to an overestimation of the intervention effect.
^6^ The statistical heterogeneity was extremely high (I² = 97.5%, P < .01), indicating that the differences in effect sizes between studies far exceeded what could be explained by random error.

^7^ The 95% confidence interval for the pooled effect SMD=3.36 was very wide (0.77 to 5.94), spanning from a "moderate effect" to an "extremely large effect," indicating very imprecise estimation of the effect size.
^8^ Among the 2 included RCTs, 1 was "high risk of bias" and 1 had "some concerns." This means 50% of the studies had serious methodological flaws. With a very small total number of studies, a single high-risk study can have a major impact on the overall reliability of the evidence, constituting what GRADE considers a "serious limitation."

^9^ The statistical heterogeneity was high (I² = 81.6%), and the test was statistically significant (P = 0.0199), indicating that the differences in effect sizes between studies far exceeded what could be explained by random error.

^10^ The 95% confidence interval for the pooled effect SMD = -0.42 was extremely wide (-5.85 to 5.00), with a width exceeding 10 standard deviation units.

^11^ The statistical heterogeneity was high (I² = 82.1%), and the test was statistically significant (P = 0.0181), indicating that the differences in effect sizes between studies were too large to be explained by random error.
^12^The 95% confidence interval for the pooled effect SMD = 0.24 was -5.21 to 5.68. The width of this interval (approximately 11 standard deviation units) is extremely abnormal.

^13^ Among the 3 RCTs included in the final analysis, 1 was "high risk of bias" and 2 had "some concerns." This means all studies had some methodological flaws, with one-third having serious flaws.
^14^ The 95% confidence interval for the pooled effect SMD = -0.58 was very wide (-2.58 to 1.43) and included the line of no effect (0).

^15^ All 3 included RCTs were rated as "high risk of bias." This means all studies had serious methodological flaws (e.g., inadequate allocation concealment, no blinding of patients or outcome assessors, etc.).

^16^ All 3 included studies were rated as "high risk of bias." This means the studies had serious methodological flaws that could systematically overestimate or underestimate the intervention effect, significantly reducing our confidence in the effect estimate.
^17^ The 95% confidence interval for the pooled effect SMD=0.61 was relatively wide (-0.05 to 1.27) and included the line of no effect (0).

^18^ Both included RCTs were rated as "high risk of bias." With a very limited evidence base, the fact that all studies had serious methodological flaws fundamentally questions the truthfulness of the effect estimate and significantly reduces confidence.

^19^ The 95% confidence interval for the pooled effect SMD = 1.40 was extremely wide (-0.23 to 3.02) and included the line of no effect (0).
